# Two Biexciton Types Coexisting in Coupled Quantum Dot Molecules

**DOI:** 10.1021/acsnano.3c03921

**Published:** 2023-07-17

**Authors:** Nadav Frenkel, Einav Scharf, Gur Lubin, Adar Levi, Yossef E. Panfil, Yonatan Ossia, Josep Planelles, Juan I. Climente, Uri Banin, Dan Oron

**Affiliations:** † Department of Physics of Complex Systems, Weizmann Institute of Science, Rehovot 7610001, Israel; ¶ Institute of Chemistry and the Center for Nanoscience and Nanotechnology, The Hebrew University of Jerusalem, Jerusalem 91904, Israel; § Departament de Quimica Fisica i Analitica, Universitat Jaume I, E-12080 Castello de la Plana, Spain; ⊥ Department of Molecular Chemistry and Materials Science, Weizmann Institute of Science, Rehovot 76100, Israel

**Keywords:** Quantum dots, Hybridization, Biexcitons, Binding energy, Single-particle spectroscopy, SPAD arrays

## Abstract

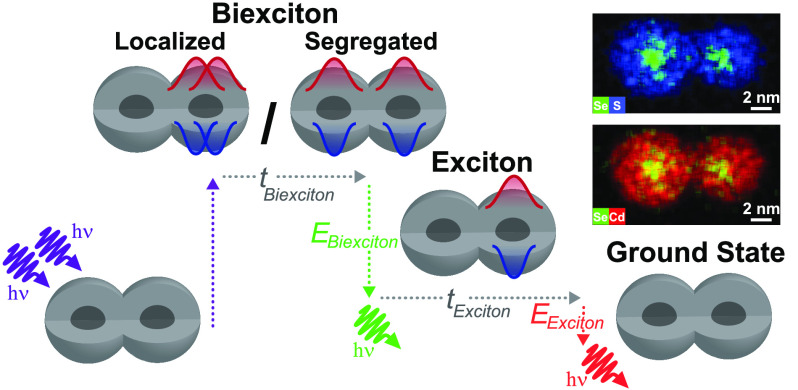

Coupled colloidal quantum dot molecules (CQDMs) are an emerging class of nanomaterials, manifesting two coupled emission centers and thus introducing additional degrees of freedom for designing quantum-dot-based technologies. The properties of multiply excited states in these CQDMs are crucial to their performance as quantum light emitters, but they cannot be fully resolved by existing spectroscopic techniques. Here we study the characteristics of biexcitonic species, which represent a rich landscape of different configurations essentially categorized as either segregated or localized biexciton states. To this end, we introduce an extension of *Heralded Spectroscopy* to resolve the different biexciton species in the prototypical CdSe/CdS CQDM system. By comparing CQDMs with single quantum dots and with nonfused quantum dot pairs, we uncover the coexistence and interplay of two distinct biexciton species: A fast-decaying, strongly interacting biexciton species, analogous to biexcitons in single quantum dots, and a long-lived, weakly interacting species corresponding to two nearly independent excitons. The two biexciton types are consistent with numerical simulations, assigning the strongly interacting species to two excitons localized at one side of the quantum dot molecule and the weakly interacting species to excitons segregated to the two quantum dot molecule sides. This deeper understanding of multiply excited states in coupled quantum dot molecules can support the rational design of tunable single- or multiple-photon quantum emitters.

Since the introduction of colloidal quantum dots (QDs) a few decades ago, their research is constantly developing, due to the intriguing quantum confinement effect that influences the electronic and optical properties as a function of the QD’s size and shape.^1,2^ QDs are impressively already widely implemented in commercial displays^3^ and are of further relevance in additional applications including lasers,^4^ light emitting diodes (LEDs),^5,6^ single photon sources,^7^ and photovoltaics.^8,9^ The extensive study in this field established synthetic means to allow for better control over the size, morphology, and surface chemistry of QDs of various semiconductor materials, enabling improved quantum yields (QYs) and tunable emission and absorption spectra.^10−12^ In recent years, further research has been carried out to synthesize more complex nanostructures with two or more coupled emission centers, thus launching the field of “nanochemistry”.^13−17^ In particular, it was demonstrated that two QDs can be fused together via a process of constrained oriented attachment, forming a coupled QD molecule (CQDM).^18−21^

As QDs are often described as “artificial atoms” due to their discrete electronic states,^22^ CQDMs are in many senses analogous to artificial molecules,^23^ manifesting hybridization of the charge carrier wave functions. For the particular case of CdSe/CdS CQDMs, electron wave functions hybridize, whereas the hole wave function is localized to the cores due to the quasi-type-II band alignment, the relatively large valence band offset between CdSe and CdS, and the heavier effective mass of the hole.^24^ Colloidal CQDMs have the potential for implementation in optoelectronics and quantum applications. Moreover, they satisfy the requirements for qubits in quantum computation^25^ and can potentially exceed the performances of epitaxial QD molecules, as they present stronger electronic coupling, observed even in room temperature, and allow flexible device production.^26^ CQDMs exhibit optical and electronic properties, which differ from their single QD building blocks as a result of the coupling.^18^ Notably, the CQDMs’ spectrum is red-shifted and broader,^20,27,28^ the absorption cross-section is modified to be doubled at high energy and smeared out near the band gap,^24^ their fluorescence decay lifetime is shorter, and their brightness is higher than their single QD constituents.^27^ The optical properties of the CQDMs depend on the width of the interfacial area between the two fused QDs, or “neck”, serving as a potential barrier. The neck can be tuned chemically during the fusion process and was found to control the extent of the coupling and thus the electronic and optical properties.^20,27^ Moreover, the joining of two light emitting centers and the increase in the volume can stabilize both charged and multiple electron–hole pairs (i.e., excitons), relative to such states in the respective single QDs, which are generally dimmed. The CQDMs structure can also accommodate different types of multiexcitonic states and relaxation pathways not present in single QDs.^27^ In the simplest case of a biexciton (BX; two excitons occupying the same CQDM), the excitons can arrange in multiple spatial configurations within these nanostructures, whereas single QDs can only accommodate a single BX spatial configuration.^27^ Moreover, CQDMs can facilitate polarization-entangled BX emission, with relevance for quantum communication.^29^

Due to exciton–exciton interactions, the BX emission in many cases is spectrally shifted from the single exciton (1X) emission.^30^ In addition, multiple recombination pathways and nonradiative processes for BXs, such as the efficient Auger recombination, reduce the fluorescence decay lifetime of the BX, relative to that of the 1X.^31^ Therefore, a better understanding of BXs in nanocrystals is crucial for their incorporation in various applications such as in lasing media, LEDs, and photovoltaics. In CQDMs, this could help reveal some of their coupling properties toward more extensive control over their multiexcitonic characteristics. However, characterization of BX emission is challenging, as they generally cannot be spectrally separated at room temperature from the neutral and charged excitonic events, due to spectral diffusion and thermal broadening.^30^ Most of the previous work in this field utilized indirect methods to characterize the BX emission. The prevalent methods were power-dependent photoluminescence and transient absorption measurements, which exhibited a large variance in results.^32−39^ Recently, direct approaches to probe BX emission events at the single-particle level were introduced, such as cascade or heralded spectroscopy.^30,40^ These recently developed methods enable the energetic and temporal detection of sequential photons, thus eliminating the ambiguity associated with indirect methods. The heralded spectroscopy technique utilizes a *spectroSPAD* system, which includes a single photon avalanche diode (SPAD) array at the output of a grating spectrometer.^30^ This system enables the postselection of cascaded BX–1X events in a time and spectrally resolved manner at room temperature. Therefore, it serves as an excellent tool for BX characterization in complex nanostructures such as CQDMs. Previous studies that utilized heralded spectroscopy used temporal photon correlation between BX and 1X emissions to measure the BX shift (Δ_*BX*_ ≡ *E*_1*X*_ – *E*_*BX*_; the difference between the spectrum peaks of the 1X and of the BX emissions) at room temperature in single CdSe/CdS/ZnS quantum dots^30^ and in CsPbBr_3_ and CsPbI_3_ perovskite nanocrystals (NCs).^41^

Herein, we explore the BX events in CQDMs and compare their properties to those of their constituent QDs, presenting an expansion of the powerful heralded spectroscopy methodology. Studying the prototypical system of CdSe/CdS core/shell CQDMs, we establish the coexistence of two BX species characterized by different lifetimes and 1X–1X interactions. Combining the experimental results with theoretical analysis, we attribute these to two BX spatial configurations. One where two holes are localized in the same QD (localized biexciton; LBX) and one where the two holes are segregated to the two constituent QDs (segregated biexciton; SBX), as illustrated at the top of Figure 1a(i).

**Figure 1:**
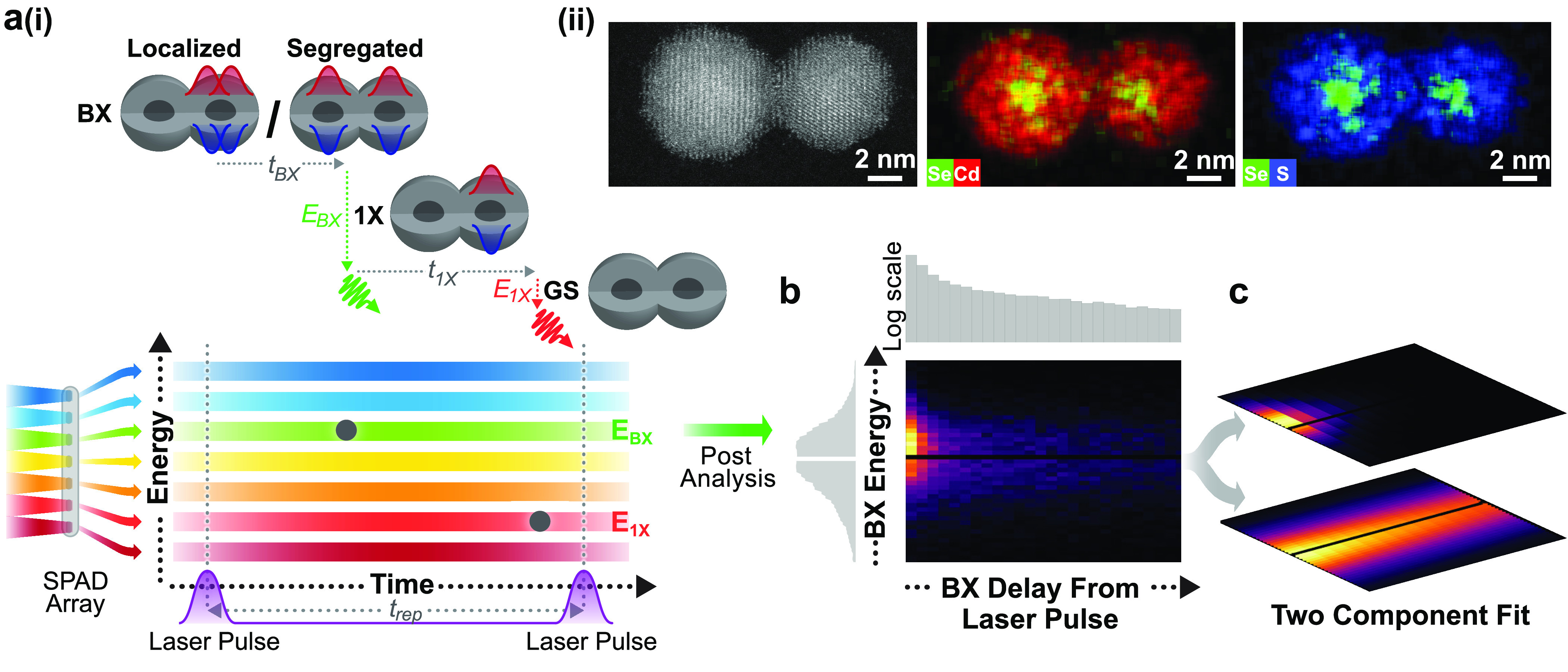
Multiple BX states in CQDMs and the heralded spectroscopy method. (a)(i) Top: Two photons are emitted sequentially by a radiative relaxation in a CQDM from a biexciton (BX) state of two possible spatial configurations to the exciton (1X) state and eventually to the ground state (GS). Bottom: Scheme of the heralded spectroscopy method that uses photon correlations to resolve the arrival time and energy of the photon pairs. Only two-photon cascades that were detected following the same excitation pulse are registered as heralded events. (ii) High-angle annular dark-field scanning transmission electron microscopy (HAADF-STEM) image and energy-dispersive spectroscopy (EDS) images of a CQDM. (b) 2D spectrum-lifetime histogram of all the postselected BX emissions from a 5 min measurement of a single CQDM. On top is the full vertical binning in logarithmic scale and to the left is the full horizontal binning of the 2D BX histogram, showcasing the BX decay lifetime and spectrum, respectively. (c) The two-component fit of the BX population in (b), each component with an independent exponential decay in time and an independent Voigt profile distribution in energy. The black horizontal line in (b) and (c) is due to a “hot” excluded pixel in the detector (see Methods section).

## Results and Discussion

The model system under study is constituted of CdSe/CdS CQDMs formed via the template approach introduced previously.^18,20^ Briefly, CdSe/CdS core/shell QDs (radius of 1.35/2.1 nm; electron microscopy characterization in Figures 1a(ii) and S1), were bound to surface-functionalized silica spheres of 200 nm in diameter, followed by controlled coverage by an additional layer of silica, which blocks the unreacted silica binding sites and partially covers the QDs’ surface, reducing the possibility to generate oligomers. Then, a molecular linker was added, followed by the addition of a second batch of the same QDs, thereby attaching to the bound QDs, forming dimers on the template. Dimers are released via selective etching of the silica spheres by hydrofluoric acid and then undergo a fusion process at a moderate temperature. Size-selective separation is performed using the controlled addition of an antisolvent, yielding a sample of the CQDMs.

In previous works that utilized heralded spectroscopy, extracting the BX emission spectrum was sufficient for a comprehensive BX characterization.^30,41^ In the current case, the analysis is extended to resolve the BX population both spectrally and temporally in order to account for the multiple BX species assumed to coexist in CQDMs. To explore BX states in CQDMs, cascaded emission events are directly probed at room temperature, extracting both temporal and spectral information simultaneously. The setup relies on exciting a single particle with a pulsed laser excitation, dispersing the emitted fluorescence by a grating spectrometer and detecting the photons (temporally and spectrally resolved) with a SPAD array detector. Occurrences of photon-pair emission detected following the same excitation pulse are postselected and treated as heralded events. Each photon within the postselected photon pairs is time- and energy-tagged according to its time and pixel of detection. The high spectral and temporal resolutions (see Methods section) enable an unambiguous temporal separation between the two detections, attributing the first arriving photon to emission from the BX state and the second photon to emission from the 1X state (Figure 1a(i) bottom).

Then, the BX population, in the form of a 2D spectrum-lifetime histogram (Figure 1b), is fitted to the sum of two independent exponentially decaying components, using a least-squares solver (Figure 1c):
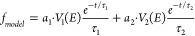
1

where *V*_*i*_(*E*) is a Voigt profile distribution in energy, i.e., over the detector’s pixels, τ_*i*_ is the component’s monoexponential decay lifetime, and *a*_*i*_ is a prefactor.

The results shown herein compare single NCs from the two samples. One is of fused CQDMs, or “fused dimers”, and one is of “nonfused dimers”, where two QDs were linked together by the same template-based procedure described above but not fused under moderate temperatures. Nonfused dimers remain connected by the molecular linker but do not feature the continuous CdS lattice, i.e., the neck, between the QDs seen in Figure 1a(ii). The dimers samples also contained single QDs, or “monomers”, that failed to attach to another QD (see Methods section and Figure S1 for further details). The monomers within the fused dimers sample were used as a reference for single QDs that underwent the same process. The photoluminescence signal from single-particle measurements was used in several further analyses, allowing nanoparticle-type classification (Figure S2), and collected under a single excitation power for all particle types (Figure S3). The additional analyses included fluorescence intensity, intensity fluctuations, decay lifetimes, and the zero-delay normalized second-order correlation of photon arrival times (*g*^(2)^(0)).^30,41^ The first two supported nanoparticle-type classification for distinguishing monomers from dimers (following ref (27)), while the *g*^(2)^(0) value was integral in revealing the nature of the NCs as quantum emitters, positioning them on the continuum between a single- and a multiple-photon emitter.

**Figure 2:**
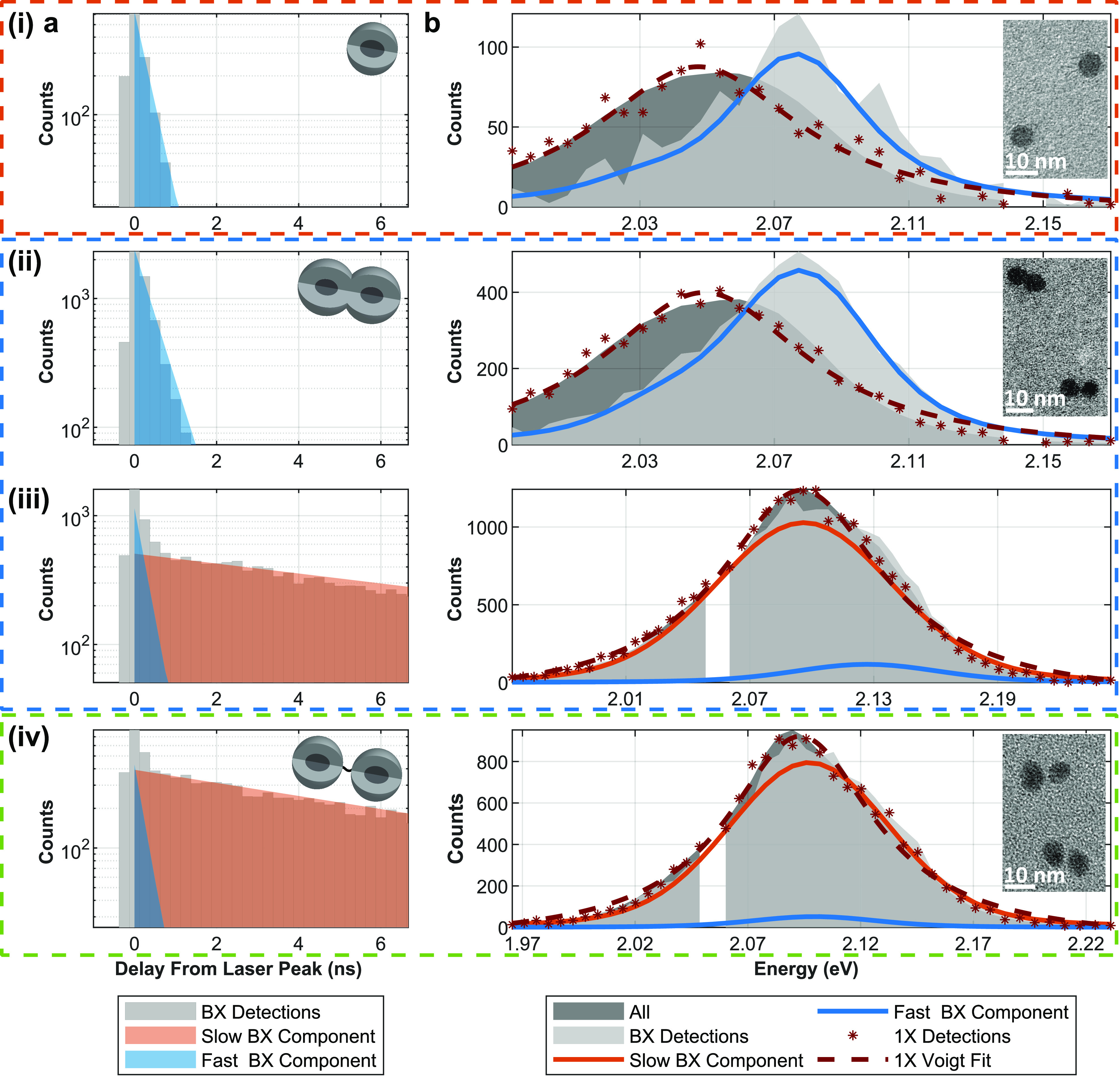
2D heralded analysis of single particles. The BX population from a 5 min measurement of (i) a monomer, a fused dimer with (ii) a high *g*^2^(0) contrast and (iii) a low *g*^(2)^(0) contrast, and (iv) a nonfused dimer. The particles feature *g*^2^(0) contrasts of approximately 0.09, 0.13, 0.37, and 0.45, respectively. Orange, blue, and green boxes distinguish between the different types of particles: monomers, fused dimers, and nonfused dimers, respectively. Schematics of the particle types are shown in the inset of (a), and transmission electron microscopy (TEM) images of the different particle types are shown in the inset of (b). The image of the fused dimers sample in panel (b)(ii) features two fused dimers that differ in the extent of fusion and filling of their interfacial area, the “neck”. (a) The bright gray bars are the full vertical binning (FVB) of the 2D BX population histogram (as the one shown in Figure 1b), showcasing the BX fluorescence decay lifetime. The blue and orange areas correspond to the FVB of the fast- and the slow-fitted BX components, respectively. A lifetime of 1 ns acts as a threshold between “fast” and “slow”. (b) The bright gray area is the full horizontal binning (FHB) of the 2D BX population histogram, showcasing the BX spectrum. The blue and orange lines correspond to the FHB of the fast- and slow-fit BX components, respectively. In red asterisks and red dashed lines are the 1X spectrum and its fitted Voigt profile, respectively. In dark gray is the normalized spectrum of all detections from the measurement. The gap in the gray areas is due to a “hot” excluded pixel in the detector (see Methods section).

Figure 2 presents representative results of the 2D heralded analysis from 5 min measurements of (i) a monomer, (ii) a fused dimer with high *g*^(2)^(0) contrast, (iii) a fused dimer with low *g*^(2)^(0) contrast, and (iv) a nonfused dimer. The left column (panel (a)) depicts the BX decay kinetics, and the right column (panel (b)) shows the BX emission spectrum, both as bright gray areas. (i) and (ii) exhibited a single exponential BX decay, whereas (iii) and (iv) exhibited a biexponential BX decay. To graphically emphasize the difference between the NCs with a single exponential decay and the ones with a biexponential feature, the fitted components were labeled as “fast” and “slow”, which refer to short and long BX decay lifetimes, respectively (blue and orange areas in panel (a), respectively). To distinguish between slow and fast decay patterns, a lifetime threshold of 1 ns was selected after preliminary results showed that monomers displayed only a subnanosecond BX decay component (Figure S4). The appearance of a >1 ns BX lifetime in dimers (Figure S5) is therefore assumed to emanate from a BX species unavailable in monomers. Cases where the 2D fit exhibited two sub-ns components might be attributed to neutral and charged BXs^42^ or simply to the additional degree of freedom in the fit. Consequently, in such cases, as in Figure 2(i,ii), the two fast components are summed together and displayed as a single fast component, which represents the observed decay.

Figure 2(i) presents a typical heralded spectroscopy characterization of a single monomer (*g*^(2)^(0) ≈ 0.09; see Figure S6). Panel (a) showcases a single subnanosecond exponentially decaying fitted component (blue area; lifetime of τ ≈ 0.3 ns), and panel (b) presents its spectrum (solid blue line). The BX shift of this component, i.e., the difference between the 1X peak (red dashed line) and the BX component’s peak, is Δ_*BX*_ = −27 ± 2 meV (all the error intervals in this work are given at 68% confidence levels of the fit). The negative BX shift (that is, a blue shift due to the 1X–1X repulsion) agrees well with a quasi-type-II band-alignment regime, in which spilling out of the electrons wave functions to the shell reduces the overlap with the holes localized in the core, and hence, the like-charges’ repulsion energies dominate over correlative attractions.^36^ The normalized spectrum of all the detections from the 5 min measurement (including single photon events) is shown in dark gray and highly matches the 1X spectrum, indicating that the overall emission is dominated by 1X emission.

Figure 2(iv) presents a typical single nonfused dimer, which in contrast to the monomer in (i), displays two different components, with lifetimes of ∼0.2 and ∼9 ns and different spectra (panels (a) and (b), respectively). The slow component (solid orange area) dominates the BX emission, with a relative contribution  of ∼97%. The fast component (solid blue line in panel (b)) features Δ_*BX*,*fast*_ = −6 ± 1 meV. The emergence of a long-lived fitted BX component with a negligible shift (Δ_*BX*,*slow*_ = −4 ± 1 meV) is naturally associated with the multiple emission centers in this single nonfused dimer.

In monomers, where the only possible BX spatial configuration is of two holes confined to one core (LBX), only a subnanosecond strongly interacting BX component is observed. Therefore, it is reasonable to attribute the reappearance of a similar fast-decaying BX component in the nonfused dimer, to LBX emission events. According to this reasoning, we assign the order-of-magnitude slower BX component to segregated BX (SBX) emission events. These assignments are validated by numerical simulations later in this section. Nonfused dimers consist of two nearly independent QDs, and thus, the SBX can be treated as two weakly interacting 1Xs, as the two holes are separated into two different cores. Hence, the BX emission from such a state is expected to resemble the 1X emission in energy but with a shorter lifetime (τ_1*X*_ ≈ 18 ns in nonfused dimers), due to multiple recombination pathways and possible nonradiative competing processes, such as energy transfer or intercore tunneling mechanisms.^18^ Moreover, in nonfused dimers, the long-lived BX is more dominant than the short-lived BX. This also supports their attribution to SBX and LBX states, respectively, as the nonradiative Auger decay dominates the LBX decay and reduces its QY. The near-zero Δ_*BX*,*slow*_ values are in agreement with the expected weak 1X–1X interaction. The small observed negative BX shift may be attributed to different quantum confinements of the constituent QDs. Bluer-emitting QDs feature shorter decay lifetimes (Figure S7a), and hence, the bluer-emitting QD of the nonfused dimer will more often emit first.

Fused dimers featured two distinct populations. Typical examples of each are seen in Figure 2(ii,iii). Case (ii) resembles the monomer in case (i) with its single subnanosecond lifetime, Δ_*BX*_ = −25 ± 1 meV, and strong photon antibunching (*g*^(2)^(0) ≈ 0.13). Case (iii) has an emerging slow component (∼11 ns lifetime), with a spectral offset (Δ_*BX*,*slow*_ = −1 ± 1 meV) resembling that of the nonfused dimer in (iv). This is accompanied by a weaker antibunching (*g*^(2)^(0) ≈ 0.37) compared to (i) and (ii). Case (iii) also shows a fast BX component that resembles the BX properties in (i) and (ii), featuring a lifetime of ∼0.3 ns and Δ_*BX*,*fast*_ = −32 ± 1 meV.

**Figure 3:**
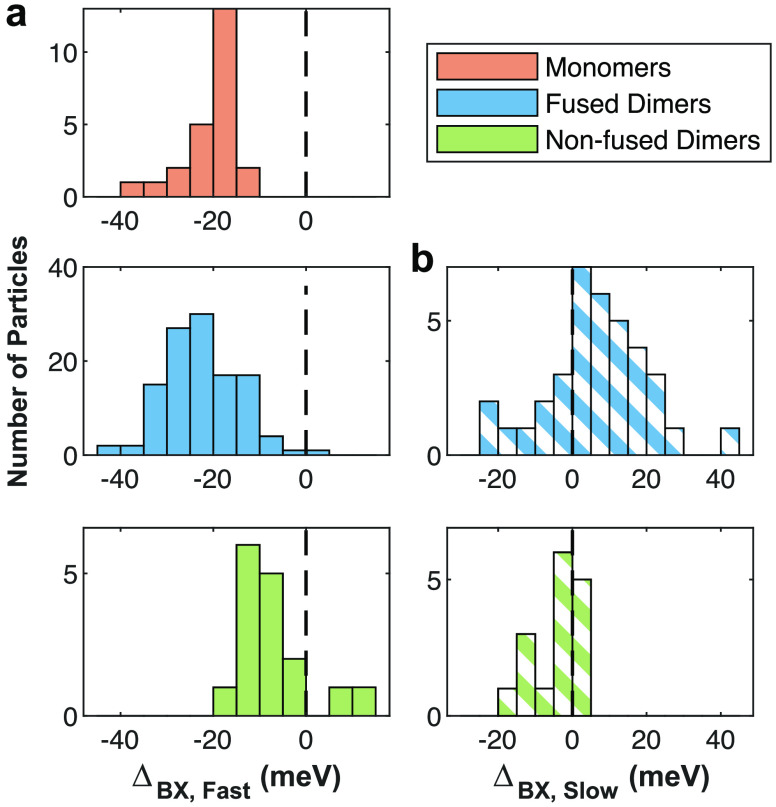
BX shifts according to particle type. BX shifts (Δ_*BX*_) of (a) the fast- and (b) the slow-fitted BX components of all the single particles according to type. Monomers and ∼68% of the fused dimers did not exhibit a component with a lifetime of 1 ns or higher and therefore do not appear in panel (b). Black dashed lines represent zero BX shift (equal energy of BX and 1X emissions).

Moving to a statistical representation measured over numerous single particles, Figure 3 displays the BX shifts of the fast and the slow components (panels (a) and (b), respectively) for all particles according to type. Monomers and ∼68% of the fused dimers presented only subnanosecond BX components and therefore do not appear in panel (b). As shown in Figure 3a, monomers and fused dimers feature a similar Δ_*BX*,*fast*_ (−21 ± 6 and −22 ± 8 meV, respectively). In nonfused dimers, the fast BX shift is weaker (Δ_*BX*,*fast*_ = −7 ± 7 meV), due to a stronger confinement effect. Unlike nonfused dimers, the monomers and fused dimers underwent ripening during the fusion process, which slightly thickened their shell, as apparent in their red-shifted emission (Figure S7b). The lower volume of nonfused dimers increases the localization of electrons, which screens the Coulombic repulsion between the holes, reducing their fast BX shift, attributed to the LBX.^39^ Indeed, the different distributions of Δ_*BX*,*fast*_ for fused and nonfused dimers are in agreement with monomers that underwent the fusion process and monomers that did not, respectively (Figure S8).

Figure 3b shows a slightly negative BX shift of Δ_*BX*,*slow*_ = −4 ± 6 meV for nonfused dimers, consistent with the previously mentioned expectation of an averaged faster emission by the bluer-emitting QD within a dimer. Notably, 32% of the fused dimers also exhibited a slow component and showed Δ_*BX*,*slow*_ = 8 ± 15 meV.

**Figure 4:**
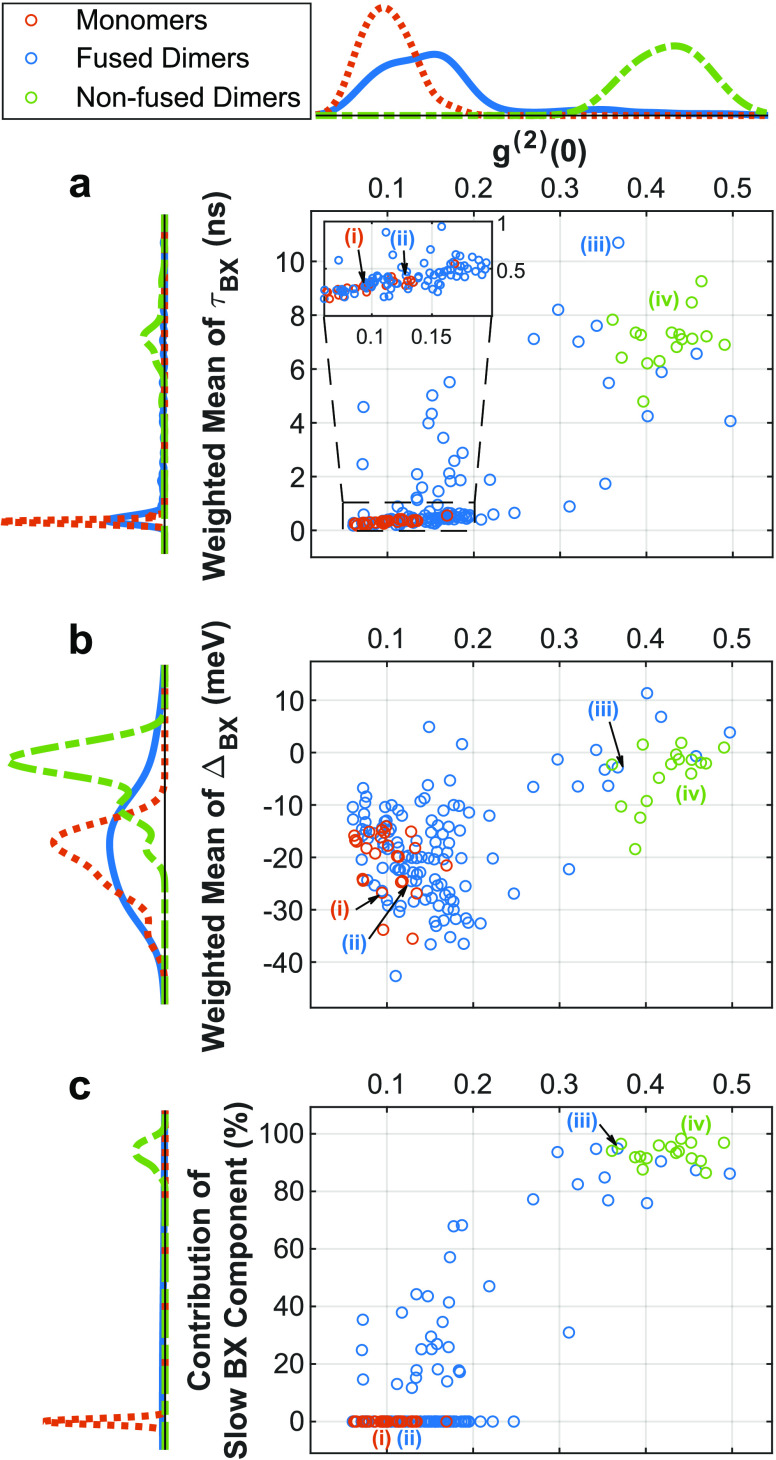
BX components as a function of *g*^(2)^(0). Weighted mean of (a) BX lifetimes and (b) BX shifts of the two fitted BX components of single particles and (c) the relative contribution of the “slow” BX component, as a function of *g*^(2)^(0), colored according to particle type. The particles shown in Figure 2 are marked with their corresponding number. Lines to the left and above the axes represent the marginal distributions as kernel density plots with colors matching the particle type. In panel (c), the particles centered at 0 contribution are those that exhibited a subnanosecond decay in both BX components.

Figure 4 shows the 2D heralded analysis of all particles as a function of the *g*^(2)^(0) contrast, indicating a single- or a multiple-photon emitter.^43^ This is with the exception of particles from the nonfused dimers sample that exhibited *g*^(2)^(0) > 0.55, which were omitted from this work. This was to avoid the possible inclusion of oligomers or charged particles (Section S3 and Figure S9). The lifetimes of the two fitted BX components and their BX shifts (i.e., the difference between the spectrum peak of the 1X and the relevant BX component) are weighted according to the component’s relative contribution . Figure 4a shows that monomers display only a fast subnanosecond BX dynamics, which agrees well with the LBX being the only available BX spatial configuration in monomers. The Auger recombination in such particles is highly efficient, leading to a high *g*^(2)^(0) contrast of 0.1 ± 0.03, classifying them as single photon emitters.

The majority of the fused dimers exhibit high *g*^(2)^(0) contrasts (<0.2) and a subnanosecond BX lifetime, which we attribute to the LBX in those systems. Their BX shift distribution overlaps that of the monomers, as is apparent in Figure 4b. Consequently, these “monomer-like” fused dimers can also be considered as single-photon emitters, yet with a larger absorption cross-section (see ⟨*N*⟩ estimation in Methods section) that increases the probability of multiexcitations.^24^ Additionally, the larger volume at the neck region allows further electron delocalization in the LBX state, which reduces the efficiency of Auger recombination and slightly increases the emission intensity and BX yield (Figure S10).

Together with increasing values of *g*^(2)^(0), the slow BX component emerges and eventually becomes the dominant one, as apparent in Figure 4c and in the increase in the weighted mean of BX lifetimes (Figure 4a) and decrease (in absolute value) in BX shifts (Figure 4b). Notably, the BX shifts of each of the two BX states do not exhibit such a correlation with *g*^(2)^(0) (Figure S11). Accordingly, we assume that the observed trends in Figure 4 result from the varying ratio of the contributions of the segregated and localized BX states.

Previous works showed that the neck thickness, which acts as a potential barrier, can control the extent of the electronic coupling, thus tuning the optical properties.^20,27^ Generally, joining two emitting centers reduces photon antibunching due to the lower rate of the nonradiative Auger recombination of multiply excited states. However, by increasing the neck width, electron wave function delocalization partially retrieves the single photon source characteristics, increasing the *g*^(2)^(0) contrast.^27^ Therefore, we suggest that the trend of a decrease in photon antibunching is correlated to a decrease in the neck size. The position of the nonfused dimers at the edge of this trend (top right corner in Figure 4a–c), with a negligible Δ_*BX*_ and a long BX lifetime, further validates the assignment of the decrease in photon antibunching as a consequence of the decrease in the neck thickness. Nonfused dimers are two monomers connected by a linker, as mentioned earlier, and exhibit *g*^(2)^(0) ≳ 0.35; therefore, they can be considered as two nearly independent monomers. This sets the monomers and nonfused dimers as the extremes on the *g*^(2)^(0) scale, with the fused dimers distributed along it according to the extent of their neck filling.^27^ Especially intriguing are fused dimers with intermediate *g*^(2)^(0) values, as they manifest an interplay between the two BX types with comparable emission of each of the BX states. They present behavior that deviates from the monomer-like regime, yet they are very different from two noninteracting QDs (Section S3). The connection between the neck thickness and *g*^(2)^(0) demonstrates a defining property of CQDMs as quantum light emitters; by controlling their neck thickness, which acts as a synthetic tunable potential barrier, it is possible to continuously alter their behavior from a single-photon emitter to a two-photon emitter.

### Quantum Mechanical Simulations

In order to establish a connection between the optical properties reported above and the morphological features of the CQDMs, we next carried out quantum mechanical simulations of the 1X and BX electronic structures in such systems. Our model is based on effective mass theory, which has successfully provided insight into the single exciton physics of CQDMs.^18,20,24,27^ Unlike in previous studies, however, we account for Coulomb interactions via a configuration interaction (CI) procedure. Compared to the self-consistent method used in earlier works,^18,24^ the CI method has the advantage of describing not only the ground state but also excited states. We shall see below that these can be relevant to understanding the optical properties at room temperature.

To gain an understanding of the CQDMs’ optical properties, we proceed in steps of increasing complexity. In the first step, we describe the BX shift in monomers through the “BX binding energy” (i.e., the difference between twice the 1X *ground* state energy and the BX *ground* state energy). In the second step, we extend the analysis to BX and 1X excited states in CQDMs that are occupied at room temperature under thermal equilibrium. At this point, the comparison of the simulated spectrum with that observed in the experiments will allow us to infer information regarding the BX relaxation dynamics and the existence of metastable excited states to explain the multiexponential BX decay observed above.

**Figure 5:**
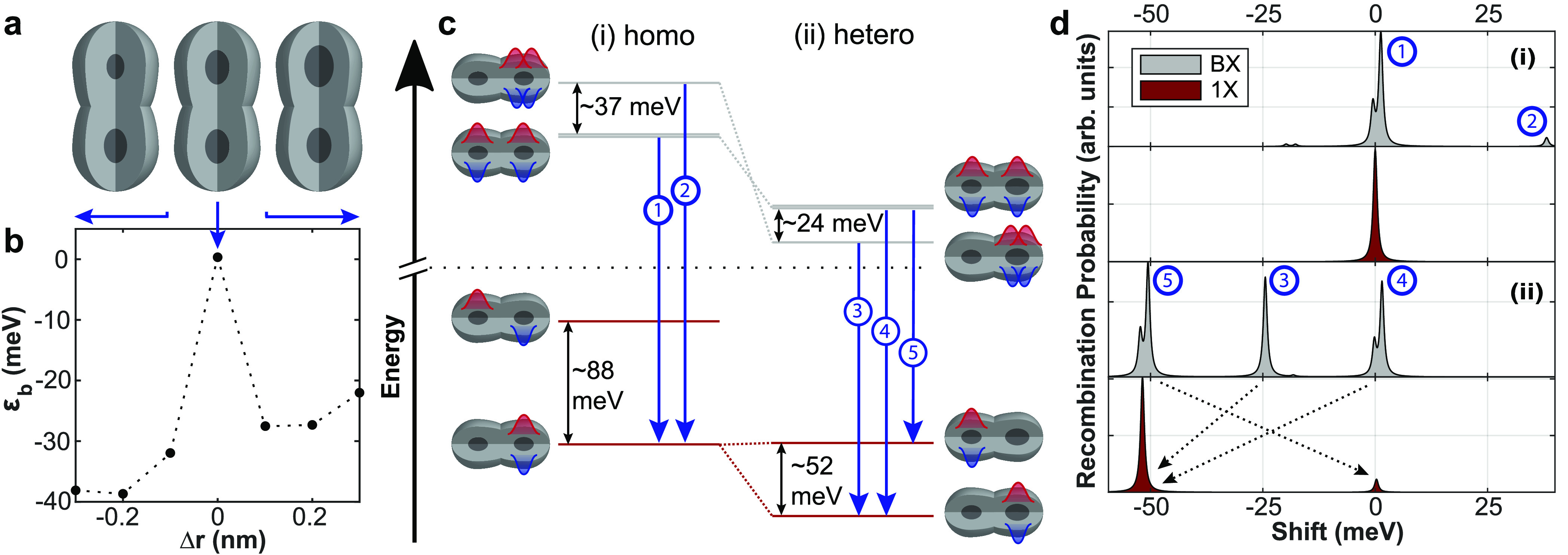
Electronic structures and calculated fluorescence spectra of BX and 1X states in CQDMs. (a) Illustration of the homodimer (center) and heterodimers (left, right) under study. (b) BX binding energy of the lowest-energy BX state as a function of the asymmetry between the sizes of the cores forming the CQDMs. Dots are calculated values, and the dotted line is a guide to the eye. A small departure from the homodimer limit (*Δr* ≈ 0.1 nm) leads to a highly negative BX shift. (c) Low-energy states of BXs and 1Xs in (i) homodimers (*Δr* = 0) and (ii) heterodimers (*Δr* = 0.2 nm). The blue arrows label the BX optical transitions, and the schematics illustrate the main charge carrier spatial configuration in the CI expansion. (d) Simulated emission spectra of the BX (bright gray) and the 1X (red) in (i) homodimers and in (ii) heterodimers, as the ones shown in (c), at *T* = 300 K. The reference energy (shift = 0 meV) is that of the 1X in the homodimers. The black dashed arrows indicate for transitions 3, 4, and 5 their respective resulting 1X state.

Here, the BX binding energy is calculated as
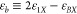
2where ε_1*X*_ and ε_*BX*_ are the ground state energies of 1Xs and of BXs, respectively. Prior to the analysis of the complex dimer system, the monomer case was simulated. The monomers are approximated as spherical core/shell particles with a total (core + shell) diameter of 6.8 nm. Negative binding energies, indicating repulsive 1X–1X interactions, in the same range as in Figure 3a, are obtained for core radii between 1.25 and 1.55 nm (Figure S14). In what follows, we consider QDs with a core radius of 1.35 nm that exhibit ε_*b*_ ≈ −35 meV, which is a slightly stronger interaction than the mean BX shift for monomers in the experimental results. Next, we studied the case of CQDMs (illustration in Figure 5a). The CdSe cores are spherical, with radii of *r*_*b*_ and *r*_*t*_ for the bottom and top cores, respectively. Each core has an ellipsoidal shell, with semiaxes *R*_*b*_ and *R*_*t*_ except for the coupling direction, where the semiaxes are *n*_*b*_ and *n*_*t*_, elongating each shell toward the other QD, thus creating an overlap between the two ellipsoids, which defines the neck filling.^24^ The central CQDM in Figure 5a illustrates a fused homodimer with *r*_*b*_ = *r*_*t*_ = 1.35 nm, *R*_*b*_ = *R*_*t*_ = 3.4 nm (according to the size of the studied constituent QDs; see Figure S1), and *n*_*b*_ = *n*_*t*_ = 7 nm, forming a thick neck region, which corresponds to a CQDM with a “rod-like” geometry (notice that a case of *n* = *R* would imply no fusion at all).

Because variations in the size of the QDs that constitute the CQDMs are likely to occur, we fix *r*_*b*_ = 1.35 nm and vary *r*_*t*_. Thus, the left and right CQDMs in Figure 5a schematically present small fluctuations in the size of the top core. Figure 5b presents the calculated BX binding energy of the lowest-energy BX state as a function of *Δr* = *r*_*t*_ – *r*_*b*_, according to eq 2. It follows from the figure that a precise homodimer (*Δr* = 0) presents a very small binding energy (ε_*b*_ ≈ 0 meV), but as soon as heterogeneity in the core sizes comes into play, the BX binding energy switches to large negative values. Thus, for cores differing only in *Δr* = ±0.1 nm, the CQDM already exhibits a BX binding energy of ε_*b*_ ≈ −30 meV. It is then clear that core size fluctuations have a major influence on the BX energetics, whereas the neck size dispersion has a much weaker influence on it (Figure S15). Note that neck size can be expected to affect the BX *dynamics* as discussed below.

To understand the origin of this seemingly bimodal distribution of BX binding energies, in Figure 5c we compare the electronic structure of a homodimer (*Δr* = 0) and a heterodimer (*Δr* = 0.2 nm). In the precise homodimer case (Figure 5c(i)), the BX ground state is the SBX, with the LBX state blue-shifted by ∼37 meV. The different energetics stems from the nature of the 1X–1X interactions in each state. In the LBX state, these are repulsive intradot interactions, much like in the monomer, whereas in the SBX, they are interdot interactions. Because 1X–1X interactions are dipole–dipole-like, they decay rapidly with distance. Interdot interactions are thus a minor effect, resulting in the SBX having about twice the energy of the 1X (i.e., ε_*b*_ ≈ 0 meV). The situation is, however, reversed in the heterodimer (Figure 5c(ii)). When one of the cores is larger than the other, the LBX with both excitons in the large QD becomes the ground state. This is because the cores are in a strong quantum confinement regime, so relaxing the confinement easily overcomes the Coulomb repulsion between excitons. For this LBX state, ε_1*X*_ and ε_*BX*_ in eq 2 are the energies of the ground 1X and BX states in the larger core, respectively, yielding ε_*b*_ ≈ – 24 meV.

For a more direct comparison with the experiments, we next study how the electronic structures of homodimers and heterodimers translate to different optical spectra. Figure 5d presents the calculated emission spectrum (assuming thermal equilibrium) of the 1X (red) and of the BX (bright gray) at room temperature for each type of CQDM, with the 1X emission in the homodimer acting as a reference point (shift = 0 meV). For the homodimer case (Figure 5d(i)), the 1X and BX spectra present a dominant peak at a similar energy. This is because at 300 K, most BXs are in the SBX state, which has ε_*b*_ ≈ 0 meV and relaxes to the direct exciton ground state (see arrow labeled as transition 1 in Figure 5c). A small peak shows up at higher energies (transition 2), which corresponds to recombination from the LBX state, but its contribution is small, as it originates from an excited state beyond thermal energy.

In the heterodimer case (Figure 5d(ii)), the 1X presents two peaks: a low-energy peak corresponding to recombination in the larger QD and a small peak at high energy corresponding to recombination in the smaller QD. The latter is small because of the scarce thermal occupation of the excited 1X state (∼52 meV above the ground state in Figure 5c). The spectrum of BX presents three relevant transitions. Transition 3 originates from the BX ground state, here the LBX. Its BX peak is blue-shifted from the main 1X transition by ∼25 meV. Transitions 4 and 5 correspond to recombination of an exciton in the smaller or larger QD, respectively. They arise from the SBX state, which can have some thermal occupation at room temperature if the core asymmetry is not large. Both transitions 4 and 5 present a double peak fine structure (splitting of ∼2 meV). This feature is a consequence of the hybridization of the electron orbitals, forming bonding and antibonding molecular states, but it is not resolved in the experiments. It is also worth noting that both transitions 4 and 5 present a similar intensity to 3, despite the SBX being a few tens of meVs higher in energy than the LBX. This is because the SBX state is highly degenerate (there are multiple ways to sort the two electrons and two holes in two QDs). This property increases the chances of room temperature occupation for SBX up to a few tens of meV above the LBX ground state, such that an SBX contribution can be expected, except in CQDMs with severe core asymmetries. According to these calculations, the spectral width of the SBX emission (transitions 4 and 5) is expected to be greater than the one of the LBX emission (transition 3), which is supported by experimental results (Figure S12).

We conclude from Figure 5d that the BX optical emission of the homodimers is governed by the SBX, which has weak 1X–1X interactions and hence emits at similar energies to the 1X. However, the BX emission energetics of heterodimers is richer, for it is governed by the LBX ground state with possible additional contributions from the SBX state. This occurs even at thermal equilibrium at room temperature and seems to account well for the observed energetic shifts of BXs for the case of heterodimers. Indeed, the heterodimer case is to be considered when interpreting the experiments, since it is unlikely that the two cores forming a CQDM would be identical at the Ångström level, which suffices to depart from the homodimer limit according to our calculations.

The simulations shed light on the experimental results. They show that core heterogeneity must be assumed, resulting in LBX as the BX ground state, at least for the majority of fused dimers. This explains the fact that most of the fused dimers exhibited a dominant LBX emission, despite its strong quenching due to Auger recombination. The calculated LBX binding energy of ε_*b*_ ≈ −25 meV also agrees with the values observed for the fast BX component shift in fused dimers, shown in Figure 3a. Nevertheless, considering the heterodimer limit, the observation of the ∼0 BX shift in some fused dimers and in the majority of the nonfused dimers (Figure 4b) is not fully explained by the calculations. Additionally, the simulations assume a thermal equilibrium between the different BX states. This would result in a single BX lifetime averaged according to the Boltzmann distribution, contrasting the observed biexponential temporal decay in this aforementioned fraction of the fused and nonfused dimers. Therefore, to explain multiple BX radiative lifetimes, we must assume metastability for the different BX states. Moreover, since the neck size is shown to have a negligible effect on the calculated energetics, we posit that it does, however, have a significant impact on the BX relaxation dynamics, which is not captured by the static simulations.

We suggest that the BX emission greatly depends on the dynamics of BX relaxation to the lower-energy BX state, which can become much faster than the radiative BX recombination when the potential barrier is low (Figure S13). Thus, assuming the case of heterodimers, as mentioned earlier, the hot generated SBX will relax with a high probability to form the lower-energy LBX in the larger QD of the pair. As the neck thickness decreases (corresponding to higher *g*^(2)^(0) in Figure 4), relaxation from an SBX to an LBX becomes slower and thus less probable, as it competes with radiative processes. Because of a higher Auger rate in the LBX state, the SBX will become the dominant emitting BX in such a case, resulting in dimers with a multicomponent BX emission characteristic. Indeed, the significant variable that changes along the decrease in photon antibunching and that agrees with its correlation with the mean BX shift in Figure 4b is the increasing ratio of SBX to LBX events (Figure 4c). Consequently, the observed behavior of BXs in CQDMs is a result of the interplay between energetics, governed by size heterogeneity, and kinetics, governed by the potential barrier.

## Conclusions

We resolve multiple biexciton species in the emission from coupled quantum dot molecules, introducing an extension to the powerful approach of heralded spectroscopy. Applying the technique to the prototypical CdSe/CdS coupled quantum dot dimers, single quantum dots, and nonfused dimers revealed the coexistence and interplay of two biexciton species. Numerical simulations and experimental results attribute the fast-decaying, strongly interacting biexciton species to localized biexcitons, where both holes are confined to the same CdSe core. The long-lived, weakly interacting biexciton species is attributed to segregated biexcitons, where the two holes reside in the two CdSe cores. The relative contribution of each species correlates with the level of antibunching, ranging from single-photon emitters to two-photon emitters, and can be tuned continuously by controlling the width of the neck barrier between the constituent quantum dots. Finally, the numerical simulations also unveil the strong dependence of the energetics of the dimers’ biexciton states on minute differences in the quantum dot core sizes, explaining the large percentage of dimers featuring single quantum dot-like behavior.

The unveiling of multiple biexciton species in coupled quantum dot molecules further demonstrates the potential of these materials as tunable and versatile quantum light emitters. Moreover, the extended heralded spectroscopy method applied here exemplifies the power and potential of this emerging spectroscopy technique to promote understanding of nanocrystal photophysics and multiple-photon quantum emitters.

## Methods

### Synthesis of CQDMs and Sample Preparation

The CdSe/CdS CQDMs were synthesized according to the protocol reported by Cui et al.,^20^ using silica nanoparticles as a template. The template was used to link CdSe/CdS monomers through a thiol group. Additional SiO_2_ was added to mask the exposed silica and immobilize the bound monomers. Introducing a second group of monomers after treating the first with a tetrathiol linker formed dimer structures, attached by the linker. Then the silica nanoparticles were etched away via a hydrofluoric acid treatment. Later, a “strong fusion process”,^20^ which includes prolonged heating, removed the linker and formed a dimer with a continuous crystalline lattice. Size-selective separation excluded a large portion of monomers, resulting in a large dimer population. A dilute solution of NCs in 2.5% poly(methyl methacrylate) in toluene was spin-cast on a glass coverslip for the single-particle measurements.

Three batches were used in this work (electron microscopy characterization in Figure S1). The first is of monomers (referred to as “pristine monomers”) that did not undergo any further synthetic process and are used mainly for reference. The second is of fused dimers that underwent the fusion process with “strong” fusion conditions (240 °C; 20 h; 5% ligands), see the inset in Figure 2(ii)(b) .^20^ Since this procedure yields not just dimers but also some monomers (and some oligomers), the NCs from the fused dimers sample were classified according to their optical properties (see Section S1 for classification details),^27^ to isolate the monomers in this sample (referred to as “monomers”). See the inset in Figure 2(i)(b) from the dimers. The last batch is of nonfused dimers, meaning pristine monomers that were linked and instead of undergoing the fusion process were only heated for 1 h at 120 °C (see inset in Figure 2(iv)(b)). This study displays results for single-particle measurements in which 400 BXs or more were detected, which amounted to 14 pristine monomers, 24 monomers out of the fused dimers sample, 116 fused dimers, and 16 nonfused dimers.

### Optical Setup

The SPAD array spectrometer is built around a commercial inverted microscope (Eclipse Ti–U, Nikon). An oil immersion objective (100, 1.3 NA, Nikon) focuses light from a pulsed laser source (470 nm, 5 MHz, LDH–P-C-470B, PicoQuant) on a single particle (QD or CQDM) and collects the emitted photoluminescence. The emitted light is then filtered through a dichroic mirror (FF484-FDi02-t3, Semrock) and a long-pass filter (BLP01-473R, Semrock). The magnified image plane (150) serves as the input for a Czerny-Turner spectrometer that consists of a 4-f system (AC254-300-A-ML and AC254-100-A-ML, Thorlabs) with a blazed grating (53-*-426R, Richardson) at the Fourier plane. At the output image plane of the spectrometer, a 512 pixel on-chip linear SPAD array is placed. Only fixed quarters of 64 pixels can participate simultaneously in the time-tagging measurement, which is done by an array of 64 time-to-digital converters (TDCs) implemented on a field programmable gate array (FPGA). The physical pixel pitch is 26.2 μm, which corresponds to a difference between neighboring pixels of ∼1.7 nm in photon wavelength or ∼5–8 meV in energy. Of the single 64-pixel segment used in this work, the 34^*th*^ pixel is a “hot” pixel and therefore omitted from all analyses. The instrument response function (IRF) of the system featured a ∼190 ps full width at half-maximum (fwhm). This response is a convolution of the excitation pulse temporal width and timing jitter of the pixels. The pixels’ dead time is ∼15 ns, and the average dark counts are ∼41 counts per second (CPS) per pixel. For further details on the experimental setup and analysis parameters, see section S2 and ref (30).

The laser illumination intensity was set to yield an average number of absorbed photons per particle per pulse (⟨*N*⟩) of ∼0.1 for pristine monomers, calculated by saturation curves of the “on” state. Using the same analysis for fused dimers yielded ⟨*N*⟩ ≈ 0.14 (see section S2 for further details).

### Quantum Mechanical Simulations

Calculations are carried within the *k* · *p* theory framework. Noninteracting (single-particle) electron and hole states are calculated with the single-band Hamiltonians and material parameters of ref (24), except for the relative dielectric constant inside the nanostructure, which is here rounded to 10. In particular, we note that the conduction band offset is 0.1 eV, which was found to provide good agreement with the experiments in earlier simulations of CQDMs.^24^ Strain and self-energy corrections are disregarded for simplicity. Many-body eigenstates and eigenenergies are calculated within a full CI method, using *CItool* codes.^44^ Coulomb integrals for the CI matrix elements, including the enhancement coming from dielectric confinement, are calculated by solving the Poisson equation with Comsol Multiphysics 4.2. The CI basis set is formed by all possible combinations of the first 20 independent-electron and 20 independent-hole spin–orbitals. Charged exciton and biexciton configurations are then defined by all possible Hartree products between the few-electron and few-hole Slater determinants, consistent with the spin and symmetry requirements. Optical spectra are calculated within the dipole approximation,^45^ assuming Lorentzian bands with a line-width of 0.5 meV. Overall, the CI model is similar to that we have used to analyze other colloidal nanostructures, where the balance between carrier–carrier interactions is a key magnitude.^46,47^

## Supporting Information Available

Characterization of the studied quantum dots, description of the analysis parameters, and supporting experimental and theoretical analyses (PDF)
