# Supplementary material for: Two Biexciton Types Coexisting in Coupled Quantum Dot Molecules
Source: ACS Nano. 2023 Jul 17;17(15):14990–5000. doi: 10.1021/acsnano.3c03921 (PMC10416571; doi:10.1021/acsnano.3c03921)
Supplement: Supplementary file 1 — nn3c03921_si_001.pdf [file nn3c03921_si_001.pdf]

Two Biexciton Types Coexisting in Coupled Quantum Dot Molecules — Supporting Information

Nadav Frenkel[1,†], Einav Scharf[2,†], Gur Lubin[1], Adar Levi[2], Yossef E. Panfil[2], Yonatan Ossia[2], Josep Planelles[3], Juan I. Climente[3,*], Uri Banin[2,*], and Dan Oron[4,*]

[1]*Department of Physics of Complex Systems, Weizmann Institute of Science, Rehovot 7610001, Israel*

[2]*Institute of Chemistry and the Center for Nanoscience and Nanotechnology, The Hebrew University of Jerusalem, Jerusalem 91904, Israel*

[3]*Departament de Quimica Fisica i Analitica, Universitat Jaume I, E-12080, Castello de la Plana, Spain*

[4]*Department of Molecular Chemistry and Materials Science, Weizmann Institute of Science, Rehovot 76100, Israel*

[†]*Equal Contributor to This Work*

[*]*Corresponding Author*

Email: climente@qfa.uji.es ; uri.banin@mail.huji.ac.il ; dan.oron@weizmann.ac.il

Abstract

This supporting information describes in greater detail the synthesis, data analysis, and system parameters used in this work, as well as additional analyses supporting the information given in the main text. Sections are brought in the order of their reference in the main text.

S1 The Studied Coupled Quantum Dot Molecule Samples

Synthetic Procedure. The synthetic procedure follows ref. 1 and is described briefly in the Methods section in the main text.

Particle Type Classification. The size-selective precipitation at the end of the synthetic dimer formation separates monomers, dimers, and multimers. Yet, the separation is not full, and monomers and oligomers are still found in the dimer samples (see Figure S1). According to transmission electron microscope (TEM) images, in the fused dimers sample ∼50% of the particles were dimers, and in the non-fused dimers sample ∼25% of the particles were dimers. Consequently, we cannot avoid single-particle measurements of all the species in these samples. Therefore, a classification procedure is required to distinguish between the single particle types. Here, we applied the classification process reported by Koley et al. and adapted it as described below.[2] We note that the strength of the 'spectroSPAD' as a comprehensive spectroscopy tool is demonstrated by the extraction of all the spectroscopic insights described below by post-processing of the same 5-min single-particle raw data collected for the heralded analysis described in the main text. Upon classifying the type of the measured single particles, it is apparent that the percentage of measured dimers exceeded their occurrence in the samples, as ∼83% of the measured particles in the fused dimers sample were dimers. At least ∼35% of the measured particles in the non-fused dimers sample were dimers (see section S3 for further insight on the non-fused dimers as nearly uncorrelated single pho-

ton sources). We attribute these statistics to selection bias, selecting brighter spots in the sample (thus avoiding most monomers) and avoiding spatially extended spots (suspected to be aggregates) during the single-particle measurements. This might have increased the fraction of the measured dimers compared with the unbiased statistics collected by electron microscopy.

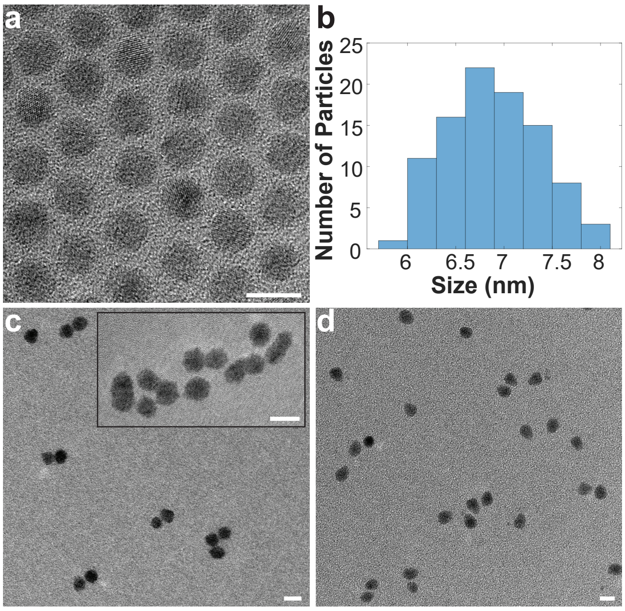

Figure S1: **Electron Microscopy Characterization of the Studied Samples. a)** High-resolution transmission electron microscopy (HR-TEM) image of "pristine monomers" (single QDs that were not exposed to fusion conditions). **b)** Size distribution of 95 pristine monomers with a mean diameter of 6.9±0.5 nm. **c)** TEM image of fused dimers. HR-TEM image in the inset showcases different extents of filling of the connecting area between the fused monomers (the neck). **d)** TEM image of non-fused dimers. The dimer samples include monomers and multimers. All scale bars are 10 nm.

The joining of two emitting centers and the different structure of dimers are manifested in different optical properties. Those differences help in distinguishing between monomers and dimers.[2] Here we present additional analyses performed to allow particle type classification.

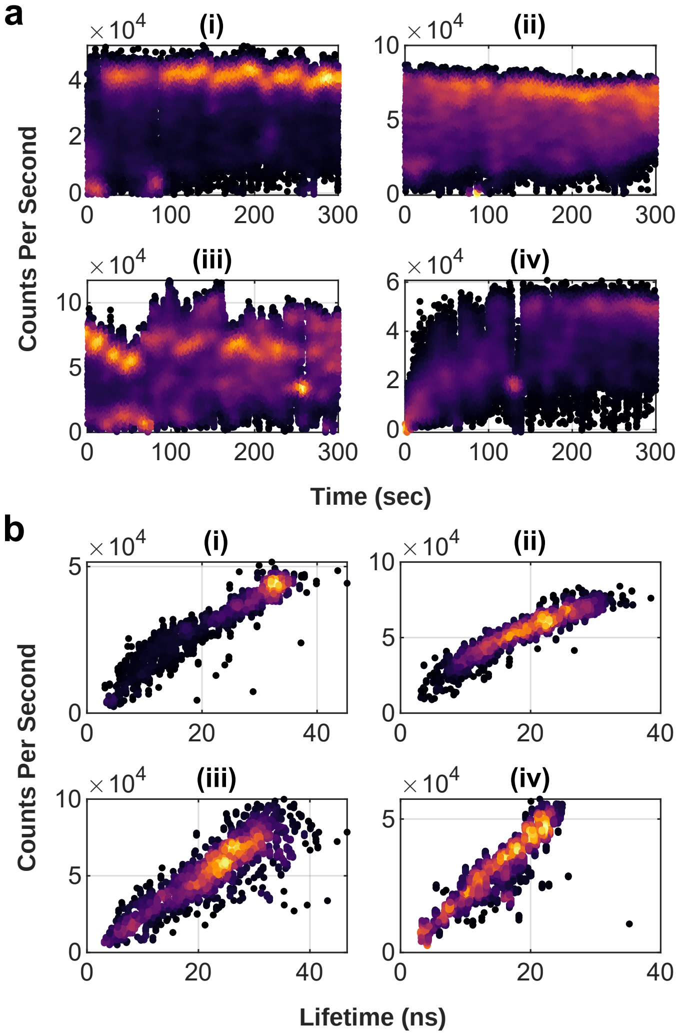

Figure S2: **Typical Optical Properties of Single Particles.** The single particles from Figure 2 in the main text are shown: (i) A monomer, a fused dimer with a (ii) high and a (iii) low $g^{(2)}(0)$ contrast, and a (iv) non-fused dimer. **a)** Fluorescence intensity fluctuation time-trace. All detections from a 5-min measurement are binned into 10 ms bins according to the global detection time. The counts per second (CPS) for each bin are then calculated. **b)** fluorescence-lifetime-intensity distribution (FLID). Each dot represents a 50 ms time-bin within the 5-min measurement. The color indicates data-point density, where brighter areas correspond to a denser population.

Examples of these are shown in Figure S2 for the single particles from Figure 2 in the main text, where (i) is a monomer, (ii) and (iii) are fused dimers with high and low $g^{(2)}(0)$ contrasts, respectively, and (iv) is a non-fused dimer. Figure S2a displays the intensity fluctuations ('blinking') of the single particles and Figure S2b presents the fluorescence lifetime-intensity distribution (FLID). In Figure S2b, detections are binned into 50 ms time bins. Each bin is assigned with an intensity value, by summing over all detections in that bin, and with an average lifetime, estimated as the temporal delay from the laser where the detections population drops by $\frac{1}{e}$. In the monomer, there is a clear 'on' state with an emission rate of $\sim 4 \cdot 10^4$ counts per second (CPS), as apparent in Figure S2a,b (i). Most photons are emitted from the 'on' state that features a relatively high count rate and a long lifetime (Figure S2b (i)). In dimers, the top count rate is higher than the $\sim 4 \cdot 10^4$ CPS of the monomer (Figure S2a,b (ii), (iii), and (iv)), which allows their identification. Figure S2b (ii), (iii), and (iv) do not feature a well-defined 'on' state, and emission is probable at different count rates. The lifetime of the frequent emitting states is shorter as well.

All of the observed differences can be associated with the larger absorption cross-section of the dimers and their larger volume compared to monomers. In monomers, Auger decay is efficient. Hence emission from charged and multi-excited states is dimmed. Most of the detections are emitted from the neutral exciton state with a high count rate and a long lifetime. In dimers, the higher count rate is attributed to the nearly two-fold absorption cross-section (see section S2).[3] The high volume of the dimers decreases the Auger rate, which increases the contribution of charged and multi-excited states. Emission from charged states, in particular, was found to be significant in dimers.[2] Accordingly, in dimers, the peak of most detections is shifted toward intermediate count rates with a shorter lifetime (Figure S2b (ii), (iii), and (iv)). The fused dimers in panels (ii) and (iii) also vary in some of their optical properties. For example, the fused dimer in panel (iii) exhibits stronger intensity fluctuations than the fused dimer in panel (ii). We later show that another differing feature between dimers is the $g^{(2)}(0)$ contrast (see Figure S6). These different properties were previously explained by variations in the potential barrier, governed by the neck thickness.[2]

The collective overview of the optical properties in Figure S2 helps to distinguish between monomers and dimers. The identification of multimers is done according to the intensity and $g^{(2)}(0)$ contrast (see section S3).

S2 System and Analyses Parameters

This section describes the measurement and analysis parameters and details some of the progress in the SPAD detector since previous accounts.[4,5]

$\langle N \rangle$ **Estimation.** To assess the saturation intensity, we follow the procedure in the Supporting Information of ref. 4. Single pristine monomers were illuminated in varying intensities, increasing every 10 seconds up to a maximal value, and then decreasing following the same steps (see Figure S3a). In order to assess the emission saturation, we plot the intensity histogram for each excitation power and identify the 'on' state peak. We use these data points to fit a saturation curve model (see Figure S3b):[6]

$$P = A(1 - e^{I/I_{sat}}) \tag{1}$$

P is the 'on' state peak and I is the excitation power. The fitted parameters are I_{sat}, which is the saturation power, and A, the asymptotic 'on' state peak. We then estimate the average number of absorbed photons per excitation pulse as $\langle N \rangle = \frac{I_{used}}{I_{sat}}$, where I_{used} is the laser intensity used in the experiment (dashed purple line in Figure S3b).

This model assumes negligible contribution by multi-excitation recombinations, which is validated by $g^{(2)}(0) \approx 0.09$ for pristine monomers under the illumination power in this study. For pristine monomers, we obtain $\langle N \rangle = 0.1 \pm 0.07$. The same procedure was done for single fused dimers and resulted in $\langle N \rangle = 0.14 \pm 0.11$. Dimers are the product of joining two monomers. Thus we expect a two-fold absorption cross-section. Yet, as discussed above, emission from charged states is significant, whereas emission from the 'on' state is less frequent. This results in an underestimation of the intensity of the 'on' state in each excitation power, which reduces the calculated $\langle N \rangle$. Still, we can set an upper limit of $\langle N \rangle \approx 0.2$ for dimers, as they consist of two monomers.

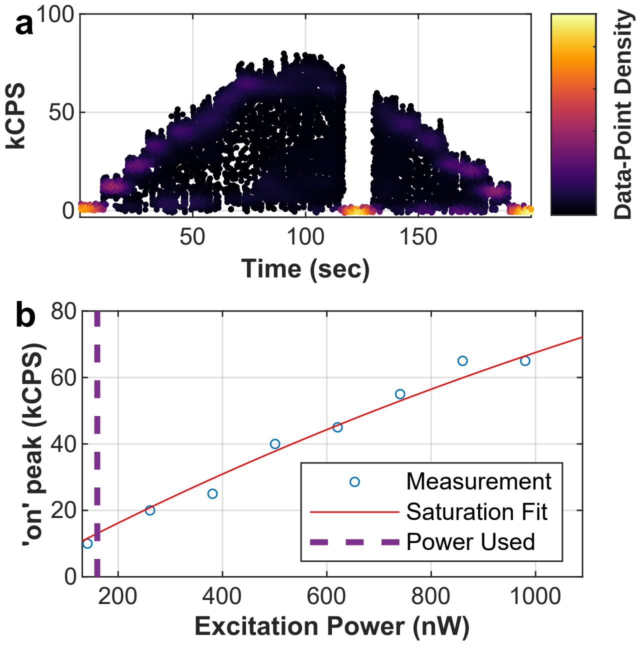

Figure S3: **Saturation Experiment of a Pristine Monomer.** **a)** Intensity fluctuation trace (shown as kilo-counts per second, i.e. kCPS) as a function of time for a single pristine monomer under varying illumination powers. The laser intensity increases in 10 seconds steps and then decreases back after reaching the maximum power. **b)** In blue circles, the intensity of the 'on' peaks at each illumination power. The red line indicates the saturation curve fit. The dashed purple line indicates the power used in the experiments of this study (160 nW). For this particle $\langle N \rangle$=0.08±0.07 (68% confidence interval).

SPAD Detector. The main difference between the experimental setup used in this work and the one depicted in refs. 4,5 is an updated pixel wiring configuration of the detector by the manufacturer. Neighboring pixels are now active during the measurement, compared with an every-other-pixel configuration in the previous version. The higher fill factor leads to a two-fold enhancement of the single-photon detection probability, which translates to a dramatic four-fold enhancement in the photon-pair detection probability. The use of neighboring pixels also increases the probability of inter-pixel optical crosstalk (a detailed description of its characterization could be found in ref. 7), which required closer attention to the corrections made. The crosstalk and dark counts contribution was calculated for each spectral-temporal bin of the 1X and BX 2D histograms and subtracted from the raw signal.

Analyses Parameters. Sequential photon emissions that were both detected after the same laser excitation pulse were registered as heralded events, as depicted in the main text. This, providing that they met the following temporal and pixel constraints. The first detected photon of the pair (BX) was constrained between -0.5 and 20 ns delay from the laser pulse peak. The lower gate is to accommodate for the instrument response function (IRF) of the system (some detections will seem to arrive before the excitation pulse due to detector jitter and excitation pulse width). The second photon of the pair (1X) was gated between 0.5 to 60 ns delay from the first photon. The non-zero lower gate serves to exclude events where the detection order is not clear, and diminish the contribution of crosstalk events (both feature temporal response corresponding to the system IRF). The upper bounds for both detections (BX and 1X) are longer than their respective lifetimes but significantly shorter than the laser pulse period (200 ns). This was chosen to lower signal loss while maintaining low dark counts contributions and ensuring both photons originated from the same excitation pulse. In addition, because of the detection dead time, mentioned in the Methods section in the main text, sequential detections in the same pixel could only occur if the photons are 15 ns apart or longer. Therefore, photon pairs that were detected at the same pixel were excluded entirely to prevent bias in favor of longer-lived photon cascades. After sifting the raw data for cascaded BX–1X events, statistical corrections for dark counts and crosstalk were applied to subtract false detections, following the scheme outlined in refs. 4,5.

Biexciton Components Distinction. Biexciton (BX) events (i.e., the first detected photon of each post-selected photon cascade) were fitted to two independent exponentially decaying components, as mentioned in the main text. The distinction of two BX sub-populations, one slowly- and one fast-decaying, was based on preliminary results for monomers and pristine monomers (see Figure S4). These showed a weighted mean BX lifetime (calculation mentioned in the main text) of the two BX components no greater than 0.6 ns. Therefore, a threshold of 1 ns ns lifetime was chosen to

distinguish between "fast" and "slow" BX components.

This distinction was made assuming that the emergence of a slow component in dimers would be due to a physical process not available in monomers. Indeed, Figure S5 shows two distinct populations of fused dimers. All fused dimers have at least one BX component with a sub-ns lifetime (blue area). For most of them, the second BX component also has a sub-ns lifetime (orange area overlapping the blue area), while for the minority, the second component has a lifetime of 1 ns or above (orange area not overlapping the blue area).

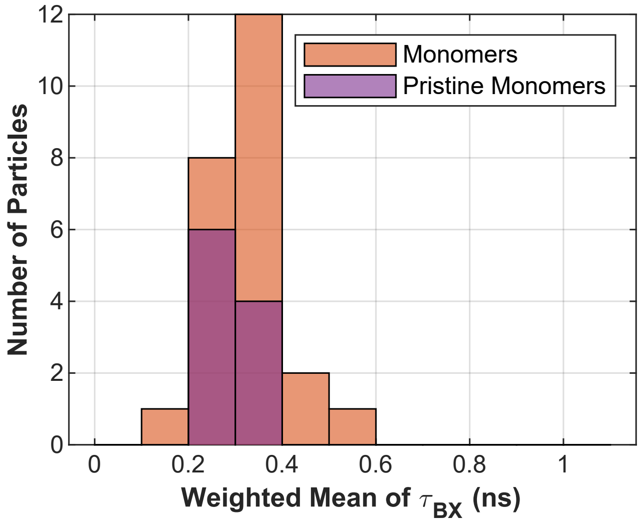

Figure S4: **BX Lifetime of Monomers and Pristine Monomers.** Weighted mean of BX lifetimes of the two BX components for monomers and pristine monomers.

S3 Supporting Analyses

This section includes further analyses performed on the single-particle level. It describes the calculation for the zero-delay normalized second-order correlation of photon arrival times ($g^{(2)}(0)$) and presents it for the single particles in Figure 2 in the main text, as an example. Then, it follows with further aggregate analyses that support the information in the main text. All supporting analyses were performed on the same raw data collected for the heralded spectroscopy and used in the main text.

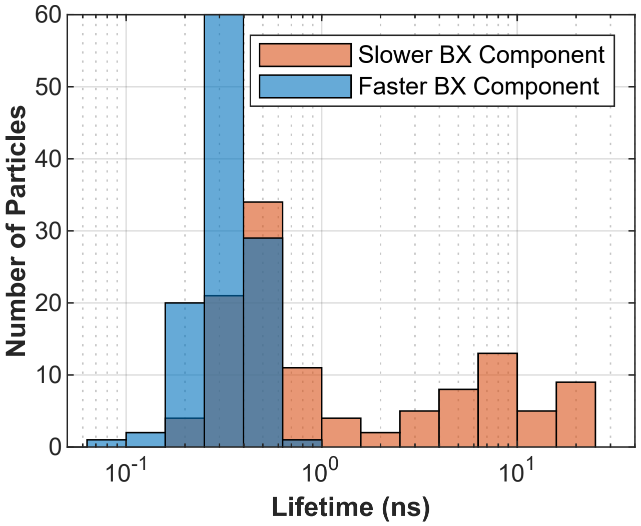

Figure S5: **Decay Lifetime of BX Components in Fused Dimers.** The lifetime distribution of the two BX components in all fused dimers, without categorizing them to "slow" and "fast" components as shown in other figures. In orange, the component with the longer lifetime out of the two and in blue, the one with the shorter lifetime.

$g^{(2)}(0)$ **Calculation.** The $g^{(2)}(0)$ was calculated and corrected for errors emanating from dark counts and inter-pixel crosstalk according to the protocol detailed in ref. 7. Briefly, each combination of SPAD array pixels pair is treated as the arms of a Hanbury Brown and Twiss photon correlation setup. Pairs of photon detections are counted according to the delay between them (τ) and binned to ∼2.5 ns bins to form the second-order correlation of photon arrival times, or $G^{(2)}(\tau)$ (after the aforementioned corrections are applied).

Figure S6 displays $G^{(2)}(\tau)$ of the single particles shown in Figure 2 in the main text. It shows a series of peaks corresponding to the laser excitation period (200 ns). The ratio between the area under the central peak and the average area under the other peaks is termed the zero-delay normalized second-order correlation of photon arrival times ($g^{(2)}(0)$). To eliminate the dominant contribution of crosstalk at shorter τ, the $G^{(2)}(0)$ bin is zeroed. To avoid a biased area ratio between peaks with and without the exclusion of the peak point, the other peak points are also zeroed and excluded from the $g^{(2)}(0)$ calculation. The particles shown display

$g^{(2)}(0)$ values of (i) ~0.09, (ii) ~0.13, (iii) ~0.37 and (iv) ~0.45.

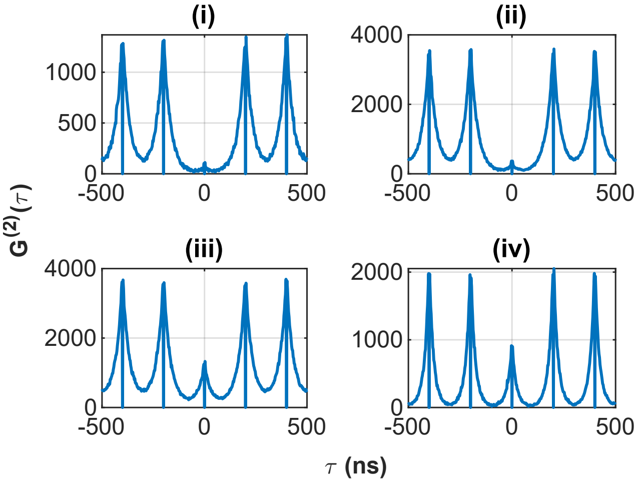

Figure S6: **Second-Order Correlation of Photon Arrival Times.** $G^{(2)}(\tau)$ of the single particles shown in Figure 2 in the main text. (i) A monomer, a fused dimer with a (ii) high and a (iii) low $g^{(2)}(0)$ contrast, and a (iv) non-fused dimer. The value of $G^{(2)}(\tau)$ indicates the number of photon pairs detected τ apart. The ratio between the area under the central peak and the average area under the other peaks is termed the zero-delay normalized second-order correlation of photon arrival times ($g^{(2)}(0)$). To eliminate the dominant contribution of crosstalk at shorter τ, the central bin of each peak is zeroed.

Comparison to Pristine Monomers. Figure S7 shows the emission spectrum peak as a function of the overall lifetime (τ_{all}), i.e., the weighted mean lifetime assessed for all detections, for each particle. All detections from a single measurement, binned according to the delay from their preceding laser pulse, are fitted to a bi-exponential decay model. The weighted mean of both lifetimes is then used to assess the overall lifetime. Figure S7a displays this plot for monomers only and shows a negative correlation between the overall lifetime and emission energy. Figure S7b shows the same plot, but for all particle types. The non-fused dimers and pristine monomers have a similar distribution in energy. This makes sense since pristine monomers are the building blocks of the non-fused dimers, both of which did not undergo fusion. Both particle types are slightly blue-shifted relative to monomers, that were exposed to the same fusion conditions as the fused dimers. This is due to the ripening process that occurs during fusion, which resulted in further shell growth and thus, a red-shift in emission energy. An additional red-shift is observed in fused dimers compared to monomers. We attribute this shift to the hybridization of the electron wave function and the additional shell volume in the neck region.

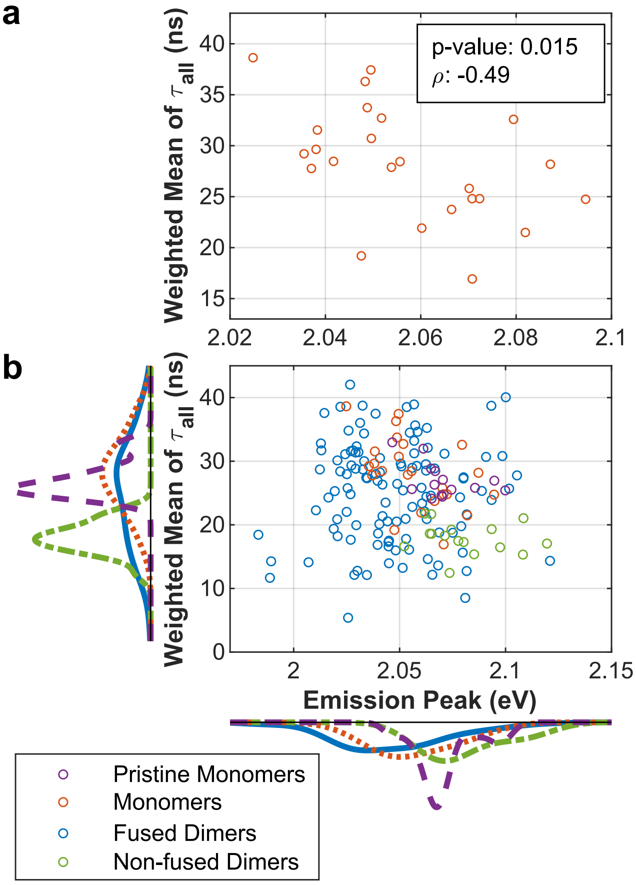

Figure S7: **Lifetime as a Function of the Peak of Emission Spectrum.** Weighted mean of the lifetimes of all detections as a function of the emission peak for **(a)** monomers only and for **(b)** all particles, colored according to particle type. p-value: p-value of Pearson's linear correlation. ρ: Pearson's linear correlation coefficient. In panel (b), lines to the left and beneath the axes represent the marginal distributions as kernel density plots, with colors matching the particle type.

Figure S7 also shows that the lifetimes of monomers and pristine monomers have similar distributions, while fused dimers exhibit slightly shorter lifetimes, possibly due to increased emission from charged and multi-excited states.[2] Non-fused dimers display a significantly shorter decay lifetime than pristine monomers. This might be due to the architecture of the non-fused dimers, increasing charge-trapping in the region between the two QDs and increasing occupation of charged exciton states, with shorter lifetimes than the neutral exciton state, thus shortening the effective decay lifetime of emission.

A further confirmation regarding the assumption of monomers' shell growth is shown in Figure S8, which compares the weighted mean of the BX shifts (Δ_{BX}) of the two BX components for monomers and pristine monomers. The weighted mean is calculated according to each component's relative contribution, as shown in Figure 4 in the main text. Monomers exhibit a slightly stronger BX shift than pristine monomers. This stronger shift agrees with the suggested shell growth in monomers, allowing electrons to delocalize further into the shell. Thus, hole–hole repulsion is given more weight, leading to further blue-shift.[8]

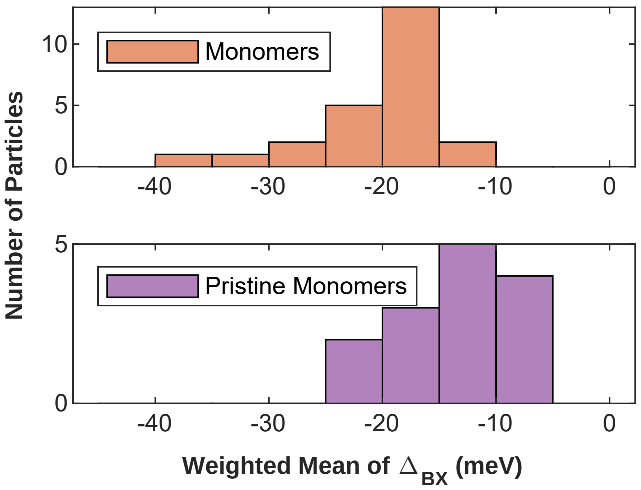

Figure S8: **BX Shifts of Monomers and Pristine Monomers.** The weighted mean of Δ_{BX} is calculated by averaging the BX shifts of the two BX components, weighting them according to their relative contribution.

Additionally, we note that the weighted mean of Δ_{BX} for pristine monomers (in Figure S8) resembles the Δ_{BX} of the fast BX component of non-fused dimers (bottom panel of Figure 3a in the main text). The fast BX component represents the LBX, which is comparable to BX states in monomers. Accordingly, we see similarities in the fast BX component's Δ_{BX} of monomers and fused dimers, which underwent fusion, and of pristine monomers and non-fused dimers, which did not undergo fusion.

$g^{(2)}(0)$ of Two Uncorrelated Photo-emitters and the Non-fused Dimers Sample. At the low excitation regime of this experiment ($\langle N \rangle \ll 1$), the photon antibunching is expected to be proportional to the BX quantum yield (QY) so that $g^{(2)}(0) \sim \frac{QY_{BX}}{QY_{1X}}$.[9] If we assume unity for the QY_{1X}, then $g^{(2)}(0) \simeq QY_{BX}$. To set an upper bound for the expected $g^{(2)}(0)$ for non-fused dimers, we consider them as two uncorrelated monomers. To be more accurate, pristine monomers are the building blocks of non-fused dimers, featuring a $g^{(2)}(0)$ distribution of 0.09±0.02, which is similar to monomers ($g^{(2)}(0)$=0.1±0.03). The calculation here is done by counting the probabilities for the different configurations of two detections following the same laser pulse ($G^{(2)}(\tau)$ central peak, or $G^{(2)}(center)$; see Figure S6) and sequential laser pulses ($G^{(2)}(\tau)$ side peaks, or $G^{(2)}(\infty)$). Considering two uncorrelated monomers, termed as emitter "A" and emitter "B", the possible configurations that contribute to $G^{(2)}(\infty)$ are A-A, B-B, A-B, and B-A, corresponding to the probability of two emissions a laser period (200 ns) apart. Each character represents an emission from the corresponding emitter ("A" or "B"). Since we assume $QY_{1X} \approx 1$, this sums up to $G^{(2)}(\infty)=4$. The possible configurations that contribute to $G^{(2)}(center)$ are AA, BB, AB, and BA, corresponding to the probability of two emissions following a single excitation pulse. The AA and BB configurations represent the probability of a BX–1X emission cascade in a monomer. Hence, they are equal to $QY_{BX,monomer} \simeq g^{(2)}(0)=0.1\pm0.03$. In contrast, the AB and BA configurations represent two non-interacting single excitons. Hence their probability is 1, as shown before. Consequently, the expected $g^{(2)}(0)$ contrast for two uncorrelated single photon emitters is $g^{(2)}(0) = \frac{G^{(2)}(center)}{G^{(2)}(\infty)} \approx \frac{2\cdot1+2\cdot0.1}{4\cdot1} = 0.55$.

We note that the non-fused dimers measured can be categorized into two distinct populations, with

$g^{(2)}(0)<0.55$ and $g^{(2)}(0)>0.55$ (see Figure S9). The first population suggests that even for the non-fused dimers, some interaction between segregated excitons may exist, which leads to enhanced antibunching. We attribute the second category of $g^{(2)}(0)>0.55$ to contribution from oligomers or charged states. The architecture of the non-fused dimers may increase charge trapping in the region between the two QDs. The charged excitons will undergo fast Auger decay and decrease the 1X and BX QYs. As the QY of the 1X is much more sensitive to charging than that of the BX,[10] $g^{(2)}(0)$ contrasts might exceed expected values, for $g^{(2)}(0)\sim\frac{QY_{BX}}{QY_{1X}}$ at the $\langle N\rangle \ll 1$ regime of this experiment (Figure S3).[9]

Figure S9 displays all particles from the non-fused dimers sample (except ones classified as monomers). Two distinct populations can be observed: one with $g^{(2)}(0)<0.5$ and another with higher values. To avoid the possible inclusion of oligomers or highly charged particles, in this work we set a threshold of $g^{(2)}(0)<0.55$, and omitted non-fused dimers with higher $g^{(2)}(0)$ values.

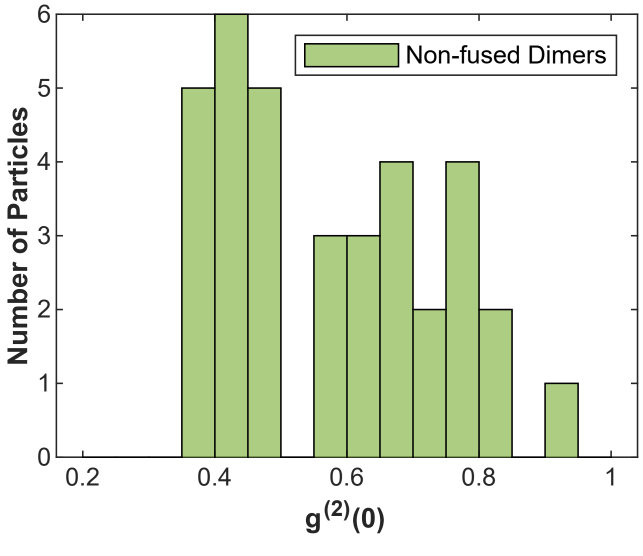

Figure S9: $g^{(2)}(0)$ **Values from the Non-fused Dimers Sample.** Photon antibunching for all particles from the non-fused dimers sample (except ones classified as monomers), without filtering for $g^{(2)}(0)<0.55$.

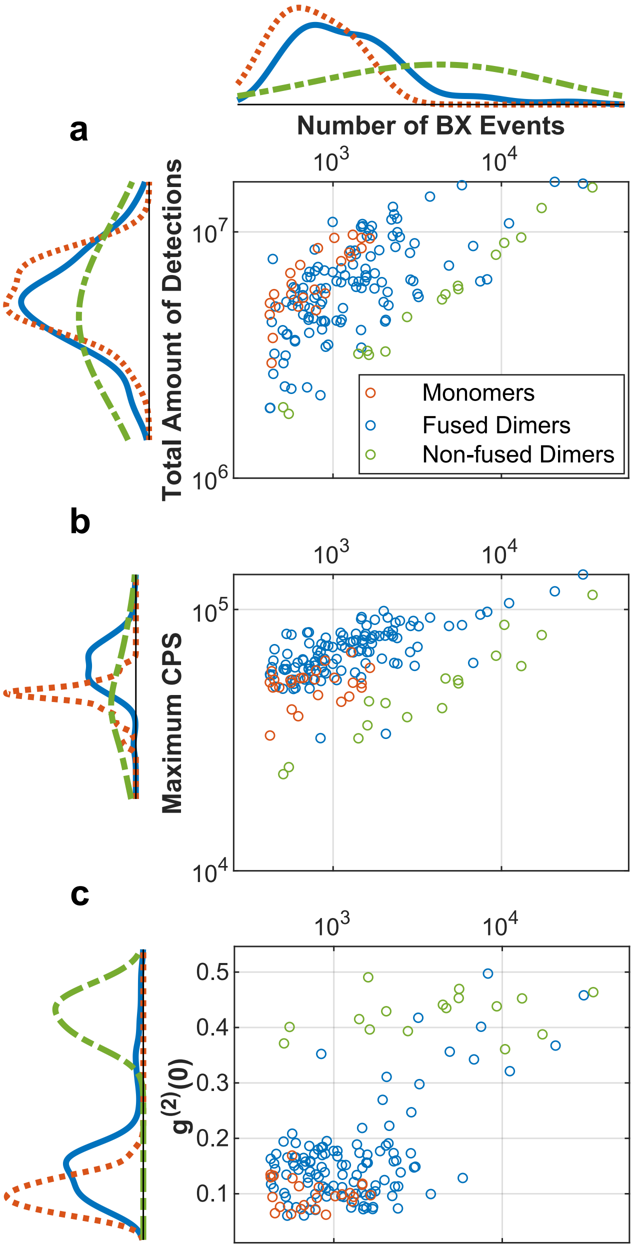

Figure S10: **Number of Heralded Events, Brightness and** $g^{(2)}(0)$**.** The number of BX events is shown as a function of **(a)** the total number of detections in a 5-minute measurement, **(b)** the maximal counts per second (CPS) in a 10 ms bin, and **(c)** the $g^{(2)}(0)$, colored according to particle type. Lines to the left and above the axes represent the marginal distributions as kernel density plots, with colors matching the particle type.

BX Detection Count. Figure S10a features the number of BX detection events as a function of the total number of detections for each 5-minute measurement. The excitation probability of single or multiple excitons follows the Poisson distribution.[11] Hence, for bright single particles, dominated by radiative exciton emission, more BX emission is expected as well. Therefore, a positive correlation is observed between the two variables. Figure S10a shows that on average, fused dimers are about as bright as monomers, whereas Figure S10b shows that upon binning the detections (10 ms bins), fused dimers exhibit higher maximal brightness (the bin with the most detections is taken as the maximum momentary brightness). These two observations support prior claims regarding the prevalence of charged states in dimers.[2] Fused dimers have a higher absorption cross-section than monomers and therefore present a brighter 'on' state (as appears in Figure S10b).[3] However, they are frequently charged, which reduces their overall counts.

As mentioned earlier, at the low excitation regime of this experiment ($\langle N \rangle \ll 1$), the photon antibunching ($g^{(2)}(0)$) is expected to be proportional to the BX quantum yield.[9] Accordingly, a strong correlation between the $g^{(2)}(0)$ and the number of BX events is observed in Figure S10c.

BX Components Shift. Figure 4 in the main text displays a variation in the mean BX shift and BX lifetime as a function of $g^{(2)}(0)$, especially for fused dimers. The fused dimers' distribution of the fast BX shift is uncorrelated with variation in $g^{(2)}(0)$ (Figure S11a). Non-fused dimers exhibit a slightly weaker fast BX shift, however, this is attributed to the increasing confinement in their constituent thinner-shell QDs, as explained in the main text. In contrast to the fast component, the BX shift of the slow BX component in fused dimers does show a stronger correlation with $g^{(2)}(0)$ (Figure S11b). Nevertheless, it shows a negative correlation with $g^{(2)}(0)$, opposite from the trend observed in Figure 4 in the main text. The slow BX component contribution, however, is positively correlated with $g^{(2)}(0)$ (Figure 4c). This trend corresponds to our suggestion in the main text that the variations in the mean BX shift and BX lifetime are carried out by the changing ratio between the contributions of the two BX components, and not by a change in the BX states' energetics.

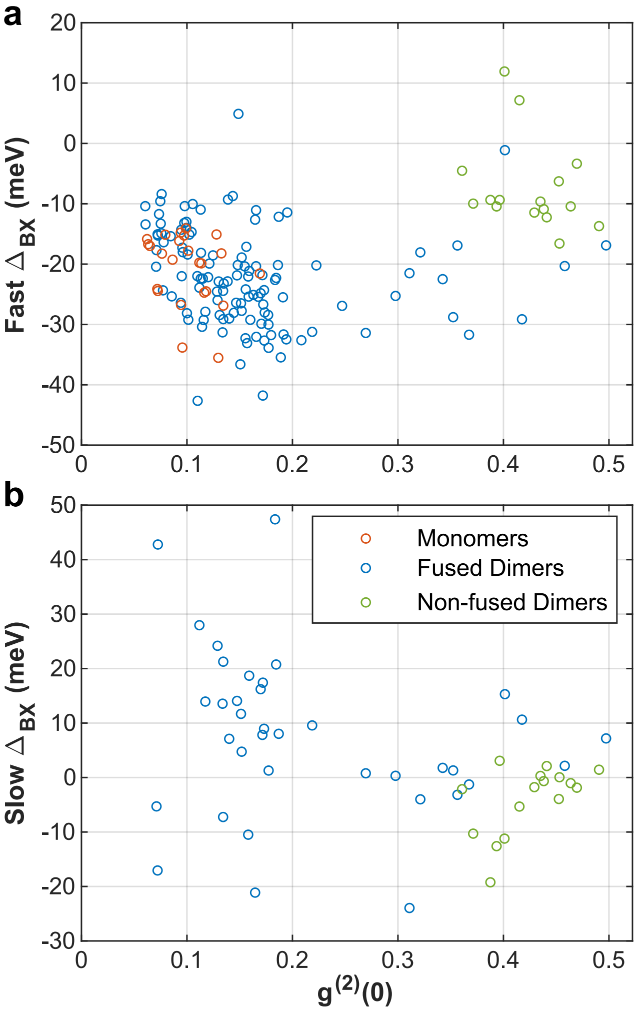

Figure S11: **BX Shifts as a Function of $g^{(2)}(0)$.** BX shifts (Δ_{BX}) of the **(a)** fast and the **(b)** slow BX components of each particle, as a function of photon antibunching. Most of the fused dimers and monomers do not appear in (b) because they do not exhibit a slow BX component at all.

BX Spectral Width. The overall BX spectral width is assessed here by fitting the whole BX population (without separation into different components) to a Voigt profile. In Figure S12, the full width at half maximum (FWHM) for each particle, according to type, is plotted against $g^{(2)}(0)$. The BX FWHM increases with $g^{(2)}(0)$, which is correlated with an increase in SBX emission over LBX, as observed in the increasing con-

tribution of the slow BX component in Figure 4c in the main text. This agrees with the expected BX spectral broadening due to an increased emission by transitions 4 and 5 (see Figure 5 in the main text) as the SBX emission increases. This is compared to a spectrally narrow BX emission expected for particles with a dominant LBX emission (monomers and most of the fused dimers), that would emit BXs mainly through transition 3.

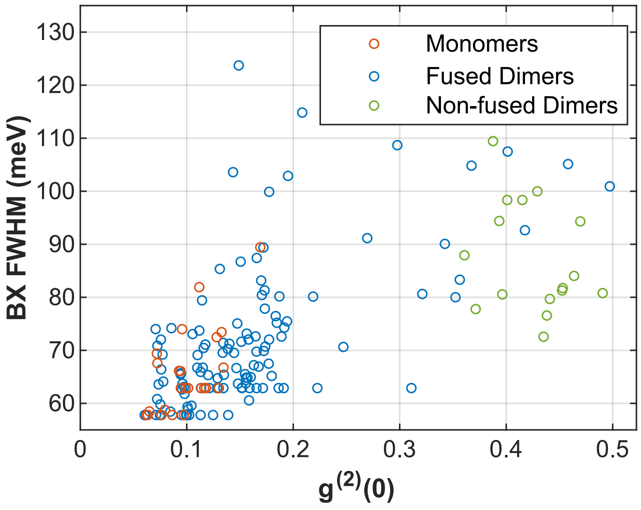

Figure S12: **BX Spectral Width.** The full width at half maximum (FWHM) of the whole BX emission, according to its fit to a Voigt profile, as a function of $g^{(2)}(0)$, colored according to particle type.

BX Initial Occupation. To study the initial occupation of the different BX species, we examine the relative amplitude of the two components, i.e., $\frac{\frac{a_i}{\tau_i}}{\sum_{i=1}^{2}\frac{a_i}{\tau_i}}$ from Equation 1 in the main text. This quantity is attributed to the relative weight of each component at $t = 0$ (compared to $\frac{a_i}{\sum_{i=1}^{2} a_i}$ which means the total contribution of each component between $t = 0$ and $t = \infty$; see Figure 4c in the main text). Following the Poisson distribution, the probabilities to initiate each of the BX states is $P_{LBX} \simeq 2P_{BX} = 2(1-(1+\langle N\rangle)e^{-\langle N\rangle})$ and $P_{SBX} \simeq P_{1X}^2 = (1-e^{-\langle N\rangle})^2$. In the $\langle N\rangle \ll 1$ regime of this experiment, their ratio is ~ 1, so we would expect that the initial excitations of the LBX and of the SBX states would be with a similar probability. This agrees well with the similar relative amplitude for the fast and slow BX components (which we attribute to the LBX and the SBX, respectively), observed in Figure S13 for non-fused dimers (relative amplitude of 40±10% for the slow component).

In comparison, all fused dimers exhibit a greater amplitude for the fast BX component (slow component relative amplitude <50%). This agrees with our assumption of exciton kinetics coming into play in our observations. As the neck size increases (i.e., $g^{(2)}(0)$ values decrease), we assume that the inter-dot transfer mechanisms become much faster than the radiative lifetime ($\tau_T \ll \tau_r$). Therefore, the transition of SBX to LBX (which we suggest is the BX ground state) is faster than our detection resolution, resulting in an apparent increased occupation of the LBX state over the SBX. In addition, for a homodimer case, the SBX state would be the ground state, which will result in a greater SBX initial occupation. All of the fused dimers and most of the non-fused dimers have a relative slow BX amplitude of <50%, showing the dominance of the LBX state, which also supports our assumption regarding the heterogeneity of all dimers.

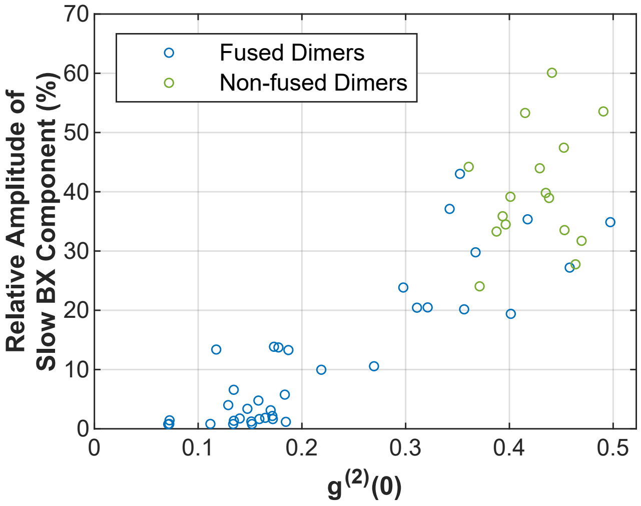

Figure S13: **Slow BX Component Initial Occupation.** The relative amplitude $\frac{\frac{a_1}{\tau_1}}{\sum_{i=1}^{2}\frac{a_i}{\tau_i}}$ of the slow BX component is shown as a function of $g^{(2)}(0)$. Only particles that featured some contribution of the slow (lifetime of >1 ns) BX component (as defined in the main text) are shown.

S4 Quantum Mechanical Simulations

Calculated Monomer BX Binding Energy. Figure S14 features the calculated BX binding energy of a monomer as a function of its core radius. The total core and shell radius is fixed to 3.4 nm while the core radius alone is varied. For the core radius in this experiment (∼1.35 nm) $\varepsilon_b \approx -34$ meV, which is a stronger 1X–1X interaction than the one observed experimentally (see Figure 3a in the main text).

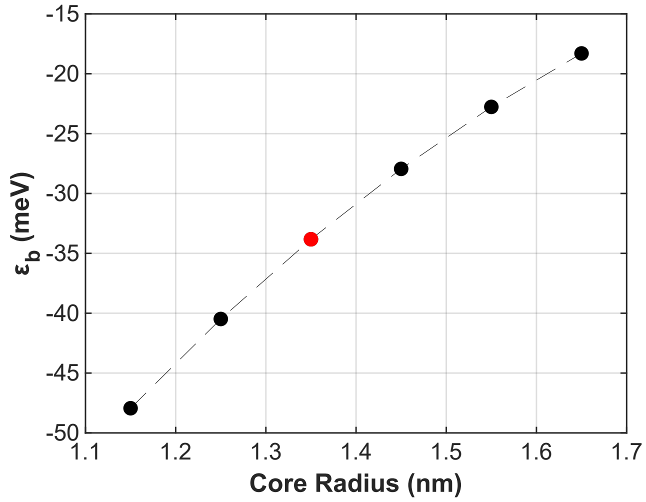

Figure S14: **Calculated Monomer BX Binding Energy.** The BX binding energy of a monomer with a fixed total radius (core + shell) of 3.4 nm as a function of the core radius. The black dots are calculated values, while the black dashed line is a guide to the eye. The red dot indicates the approximate core size of the particles studied experimentally.

Neck Size Effect on BX Binding Energy. Figure S15 exhibits the calculated effect the neck size has on the BX binding energy in homodimers. The BX binding energy is always close to zero, which is a result of the weak inter-dot 1X–1X interaction in the SBX state (i.e., the lower-energy BX state in homodimers; see main text). The neck size has a minor influence on the electronic structure because the wave functions of both the electrons and holes are mostly localized in or around the respective cores. The neck was found to have a negligible effect on the BX binding energy in the heterodimer case as well. Notice that the y-axis units in Figure S15b are in meVs, displaying a change in ε_b of <1 meV while varying from a dimer with almost no connecting neck (n=4 nm) to a one with a 'rod-like' geometry (n=7 nm).

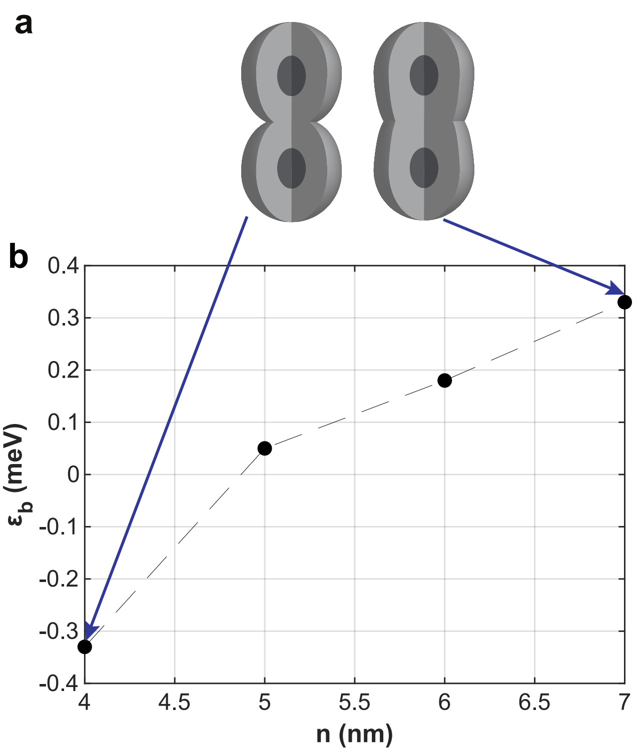

Figure S15: **Neck Size Effect on BX Binding Energy. a)** Illustrations of homodimers with a small (left) and a large (right) neck size. **b)** The BX binding energy of a homodimer is shown as a function of n, which is the semi-axis of the ellipsoidal shell in the coupling direction of the dimer. An increase in n corresponds to a larger neck size, as explained in the main text. The black dots are calculated values, while the black dashed line is a guide to the eye.

References

[1] Cui, J.; Koley, S.; Panfil, Y. E.; Levi, A.; Ossia, Y.; Waiskopf, N.; Remennik, S.; Oded, M.; Banin, U. Neck Barrier Engineering in Quantum Dot Dimer Molecules via Intraparticle Ripening. *Journal of the American Chemical Society* **2021**, *143*, 19816–19823.

[2] Koley, S.; Cui, J.; Panfil, Y. E.; Ossia, Y.; Levi, A.; Scharf, E.; Verbitsky, L.; Banin, U. Photon correlations in colloidal quantum dot molecules controlled by the neck barrier. *Matter* **2022**, *5*, 3997–4014.

[3] Panfil, Y. E.; Shamalia, D.; Cui, J.; Koley, S.; Banin, U. Electronic coupling in colloidal quantum dot molecules; the case of CdSe/CdS core/shell homodimers. *Journal of Chemical Physics* **2019**, *151*.

[4] Lubin, G.; Tenne, R.; Ulku, A. C.; Antolovic, I. M.; Burri, S.; Karg, S.; Yallapragada, V. J.; Bruschini, C.; Charbon, E.; Oron, D. Heralded Spectroscopy Reveals Exciton-Exciton Correlations in Single Colloidal Quantum Dots. *Nano Letters* **2021**, *21*, 6756–6763.

[5] Lubin, G.; Yaniv, G.; Kazes, M.; Ulku, A. C.; Antolovic, I. M.; Burri, S.; Bruschini, C.; Charbon, E.; Yallapragada, V. J.; Oron, D. Resolving the Controversy in Biexciton Binding Energy of Cesium Lead Halide Perovskite Nanocrystals through Heralded Single-Particle Spectroscopy. *ACS Nano* **2021**, *15*, 19581–19587.

[6] Teitelboim, A.; Oron, D. Broadband Near-Infrared to Visible Upconversion in Quantum Dot–Quantum Well Heterostructures. *ACS Nano* **2015**, *10*, 446–452.

[7] Lubin, G.; Tenne, R.; Antolovic, I. M.; Charbon, E.; Bruschini, C.; Oron, D. Quantum correlation measurement with single photon avalanche diode arrays. *Opt. Express* **2019**, *27*, 32863–32882.

[8] Oron, D.; Kazes, M.; Banin, U. Multiexcitons in type-II colloidal semiconductor quantum dots. *Physical Review B - Condensed Matter and Materials Physics* **2007**, *75*.

[9] Nair, G.; Zhao, J.; Bawendi, M. G. Biexciton quantum yield of single semiconductor nanocrystals from photon statistics. *Nano Letters* **2011**, *11*, 1136–1140.

[10] Xu, W.; Hou, X.; Meng, Y.; Meng, R.; Wang, Z.; Qin, H.; Peng, X.; Chen, X. W. Deciphering Charging Status, Absolute Quantum Efficiency, and Absorption Cross Section of Multicarrier States in Single Colloidal Quantum Dots. *Nano Letters* **2017**, *17*, 7487–7493.

[11] Peterson, J. J.; Nesbitt, D. J. Modified power law behavior in quantum dot blinking: A novel role for biexcitons and auger ionization. *Nano Letters* **2009**, *9*.
